# PoRVA G9P[23] and G5P[7] infections differentially promote PEDV replication by reprogramming glutamine metabolism

**DOI:** 10.1371/journal.ppat.1012305

**Published:** 2024-06-21

**Authors:** Haixin Liu, Haolun Tian, Pengcheng Hao, Huimin Du, Kun Wang, Yudong Qiu, Xiangrui Yin, Nana Wu, Qian Du, Dewen Tong, Yong Huang

**Affiliations:** 1 College of Veterinary Medicine, Northwest A&F University, Yangling, China; 2 Engineering Research Center of Efficient New Vaccines for Animals, Ministry of Education of the People’s Republic of China, Yangling, China; 3 Key Laboratory of Ruminant Disease Prevention and Control (West), Ministry of Agriculture and Rural Affairs, Yangling, China; 4 Engineering Research Center of Efficient New Vaccines for Animals, Universities of Shaanxi Province, Yangling, China; University of Michigan, USA, UNITED STATES

## Abstract

PoRVA and PEDV coinfections are extremely common in clinical practice. Although coinfections of PoRVA and PEDV are known to result in increased mortality, the underlying mechanism remains unknown. Here, we found that PoRVA infection promoted PEDV infection *in vivo* and *in vitro* and that PoRVA G9P[23] (RVA-HNNY strain) enhanced PEDV replication more significantly than did PoRVA G5P[7] (RVA-SXXA strain). Metabolomic analysis revealed that RVA-HNNY more efficiently induced an increase in the intracellular glutamine content in porcine small intestinal epithelial cells than did RVA-SXXA, which more markedly promoted ATP production to facilitate PEDV replication, whereas glutamine deprivation abrogated the effect of PoRVA infection on promoting PEDV replication. Further studies showed that PoRVA infection promoted glutamine uptake by upregulating the expression of the glutamine transporter protein SLC1A5. In SLC1A5 knockout cells, PoRVA infection neither elevated intracellular glutamine nor promoted PEDV replication. During PoRVA infection, the activity and protein expression levels of glutamine catabolism-related enzymes (GLS1 and GLUD1) were also significantly increased promoting ATP production through glutamine anaplerosis into the TCA cycle. Consistent with that, siRNAs or inhibitors of GLS1 and GLUD1 significantly inhibited the promotion of PEDV replication by PoRVA. Notably, RVA-HNNY infection more markedly promoted SLC1A5, GLS1 and GLUD1 expression to more significantly increase the uptake and catabolism of glutamine than RVA-SXXA infection. Collectively, our findings illuminate a novel mechanism by which PoRVA infection promotes PEDV infection and reveal that the modulation of glutamine uptake is key for the different efficiencies of PoRVA G9P[23] and PoRVA G5P[7] in promoting PEDV replication.

## Author summary

All phases of the viral life cycle are heavily dependent on host metabolism, thus virtually all viruses actively reprogram host metabolic networks to support their replication and spread. However, in the case of PoRVA and PEDV coinfection, the impact of host cell metabolic reprogramming induced by PoRVA infection on PEDV replication has not been reported. Herein, we demonstrated that PoRVA infection promotes PEDV replication by targeting glutamine metabolism in porcine intestinal epithelial cells. PoRVA infection induces increased expression levels of SLC1A5, GLS1, and GLUD1, which enhance glutamine uptake, TCA cycle flux, and ATP production. Notably, we found that PoRVA G9P[23] infection more effectively promoted PEDV replication than PoRVA G5P[7] infection by significantly increasing glutamine uptake and anaplerosis into the TCA cycle.

## Introduction

Rotaviruses (RVs) and coronaviruses (CoVs) are RNA viruses that can infect humans and other mammals, and pose an ongoing threat to public health [1]. Because of their worldwide distribution and the ability to spread efficiently, infected animals tend to considerable morbidity and mortality. RVs are a significant cause of acute viral gastroenteritis in both children and production animals, including pigs [2]. RVs are non-enveloped double-stranded RNA viruses that contain 11 segments of dsRNA encoding non-structural proteins (NSP1 to NSP5/6) and viral structural proteins (VP1 to VP4, VP6, and VP7). RVs are classified into 11 distinct groups, RV A-D F-L, which are further subdivided into distinct G/P genotypes based on the molecular characteristics of VP7 and VP4 [3]. Currently, RV A, B, C, and H have been identified in pigs; among them, RVA is considered the most common pathogenic RV species in pigs worldwide [4,5]. Porcine epidemic diarrhea virus (PEDV), a member of the *Alphacoronavirus* within the *Coronaviridae* family, is a highly infectious pathogen that causes epidemics in swine herds. This virus is a large-enveloped virus, with a single-stranded positive-sense RNA genome of approximately 28 kb containing at least 6 open reading frames (ORFs). PEDV has become a globally emerging and re-emerging epizootic swine enteric virus that causes massive economic losses in the swine industry, with high mortality in piglets [6]. The clinical signs of PEDV infection include vomiting, diarrhea, and dehydration, which can reach 100% mortality in 1- to 3-day-old piglets [7].

Almost invariably under natural circumstances pigs may be infected by multiple viruses, and coinfections of multiple viruses are thought to have a greater impact on disease outcome than single virus infections [8,9]. Coinfecting viruses can interact among themselves and with host symbionts to utilize host resources or modulate host immune responses [10]. Swine gastrointestinal diseases caused by viral infection have long been among the most serious diseases in the pig industry, and they have become increasingly serious in recent years. In particular, coinfection with multiple gastrointestinal viruses not only aggravates the clinical signs of enteric diseases but also increases the difficulty of diagnosing and preventing the disease [11]. To date, PoRV, PEDV, and other common swine enteric viruses have caused considerable economic losses to the pig industry in China [12], and coinfection with these swine enteric viruses has been previously reported in different provinces in China [12–14].

During viral infections, a wide variety of divergent viruses have evolved mechanisms that manipulate cellular metabolic pathways to obtain sufficient availability of biomolecules and energy for their survival and replication [15]. Because viruses are obligate intracellular parasites, each step of viral infection is carried out in ‘virus factories’, which are created from virus-induced metabolically reprogrammed cells. It is not surprising that viral infection triggers metabolic reprogramming in host cells to meet their biosynthetic demands for optimal virus production [16]. Different types of viruses exploit several strategies to hijack cellular nutrient resources, thereby determining viral pathogenesis and the host response. For example, SARS-CoV-2 infected individuals may have higher blood glucose and fatty acid concentrations and abnormalities in amino acid metabolism [17]. In SARS-CoV-2-infected cells, viral nonstructural protein 14 (NSP14) promotes the succinylation of several key enzymes in the TCA cycle that interact with SIRT5, thereby inhibiting cellular metabolic pathways [18]. Rhinovirus infection rapidly enhances the expression of enzymes responsible for glucose utilization and uptake, leading to a highly anabolic state [19]. Recent studies have shown that giant virus genomes contain numerous metabolic genes involved in energy production [20]. The metabolic genes encoded by giant viruses also manipulate host metabolism, by expanding the catalytic capabilities of host cells, favoring viral replication [21]. Although virus-triggered metabolic alterations are arguably a hot research frontier, the molecular mechanisms underlying virus-induced metabolic reprogramming remain mostly unknown.

Outbreaks of swine enteric diseases are frequently caused by multiple enteric viruses, and the coexistence of several viruses in the gut aggravates clinical signs and increases the difficulty of disease prevention and control [22]. In the present study, we investigated the mechanism by which PoRVA infection promotes PEDV replication by modulating glutamine metabolism in small intestinal epithelial cells, and identified the predominant host proteins responsible for glutamine uptake and conversion during the PoRVA-induced reprogramming of glutamine metabolism. Moreover, we investigated the differences between the effects of PoRVA G9P[23] and PoRVA G5P[7] on the reprogramming of glutamine metabolism and the promotion of PEDV replication. This study will improve our understanding of the mechanisms by which PoRVA infection induces metabolic reprogramming to promote PEDV replication, as well as the reasons for the differences among PoRVA subtypes in promoting PEDV replication.

## Results

### PoRVA infection promotes PEDV infection in pigs

**Fig 1:**
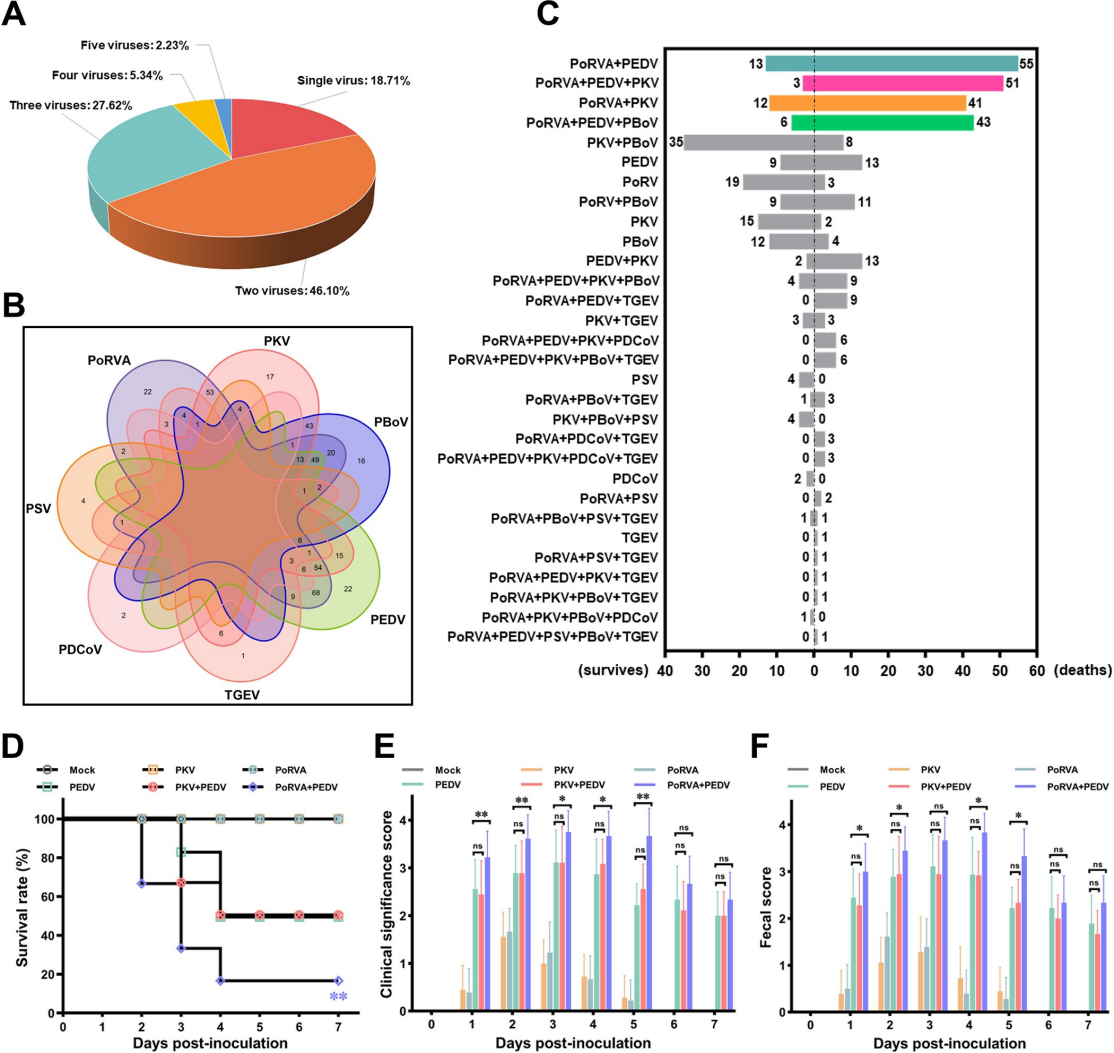
PoRVA infection enhances the pathogenicity of PEDV in cases coinfected with PoRVA and PEDV. **(A)** The patterns of enteric virus infection in virus-positive cases. **(B)** Venn diagram showing the patterns of virus coinfection and the corresponding number of cases. **(C)** Comparative survival analysis of cases with different infection patterns. PoRVA: porcine rotavirus Group A; PEDV: porcine epidemic diarrhea virus; PKV: porcine kobuvirus; PBoV: porcine bocavirus; TGEV: transmissible gastroenteritis virus; PDCoV: porcine deltacoronavirus; PSV: porcine sapelovirus. **(D-F)** PoRVA aggravated the pathogenicity of PEDV in piglets. The piglets were infected with PoRVA (4 mL, 10^5^ PFU/mL), PKV (4 mL, 10^5^ PFU/mL), or mock (4 mL, DMEM) for 12 h and than inoculated with 4 mL of PEDV (10^5^ PFU/mL). The survival curves (D), clinical significance scores (E), and fecal scores of the piglets (F) in each group were continuously monitored and recorded until the end of the experiment at 7 dpi. ns, not significant, * *P* < 0.05, ** *P* < 0.01 (compared with the PEDV infection group).

Swine gastrointestinal diseases are usually caused by various enteric viruses, and coinfection with enteric viruses may exacerbate disease outcomes and seriously threaten herd health [23]. Herein, we first conducted an epidemiological survey of enteric viruses in Midwest China. A total of 582 samples were collected from 34 pig farms in four provinces to detect the positivity rates of common viruses from October 2020 to December 2022 (S1A Fig). We found that PoRVA (54.81%, 319/582) and PEDV (42.44%, 247/582) were the most common viruses causing diarrhea in the surveyed pigs (S1B Fig). Analysis of the infection patterns of enteric viruses revealed that the proportion of diarrhea caused by coinfection with multiple viruses was markedly greater than that caused by infection with a single virus (Fig 1A). Among multiple virus positive cases, the proportion of coinfection of two viruses was the largest (56.71%, 207/365) (Fig 1B), and coinfection with PEDV and PoRVA was most common in double virus-positive pigs (32.85%, 68/207) (Fig 1B and 1C). In addition, in PoRVA-positive cases, coinfection with PEDV was the most frequent infection pattern (210/319) (S1C Fig), and PEDV infection was potentially associated with PoRVA infection in clinic (S1D Fig).

Notably, among multiple virus positive cases, coinfection with PoRVA and PEDV led to a greater mortality rate in infected pigs (Fig 1C). Thus, we speculated that PoRVA infection enhances PEDV infectivity and pathogenicity. The effects of mock infection, PoRVA infection, and PKV (Porcine Kobuvirus) infection on the pathogenicity of PEDV infection were compared by piglet infection experiments. The results showed that PoRVA infection remarkably aggravated the clinical signs and increased the mortality of piglets when challenged with same doses of PEDV infection, whereas PKV infection had no significant effects on the infectivity and pathogenicity of PEDV in the piglet infection experiments (Fig 1D–1F). Taken together, these results demonstrate that coinfection with PoRVA and PEDV is the most common infection pattern in the field clinic and suggest that PoRVA infection can exacerbate PEDV infection in piglets.

### PoRVA G9P[23] genotype strain more strongly promotes PEDV replication to enhance PEDV pathogenicity than PoRVA G5P[7] genotype strain

**Fig 2:**
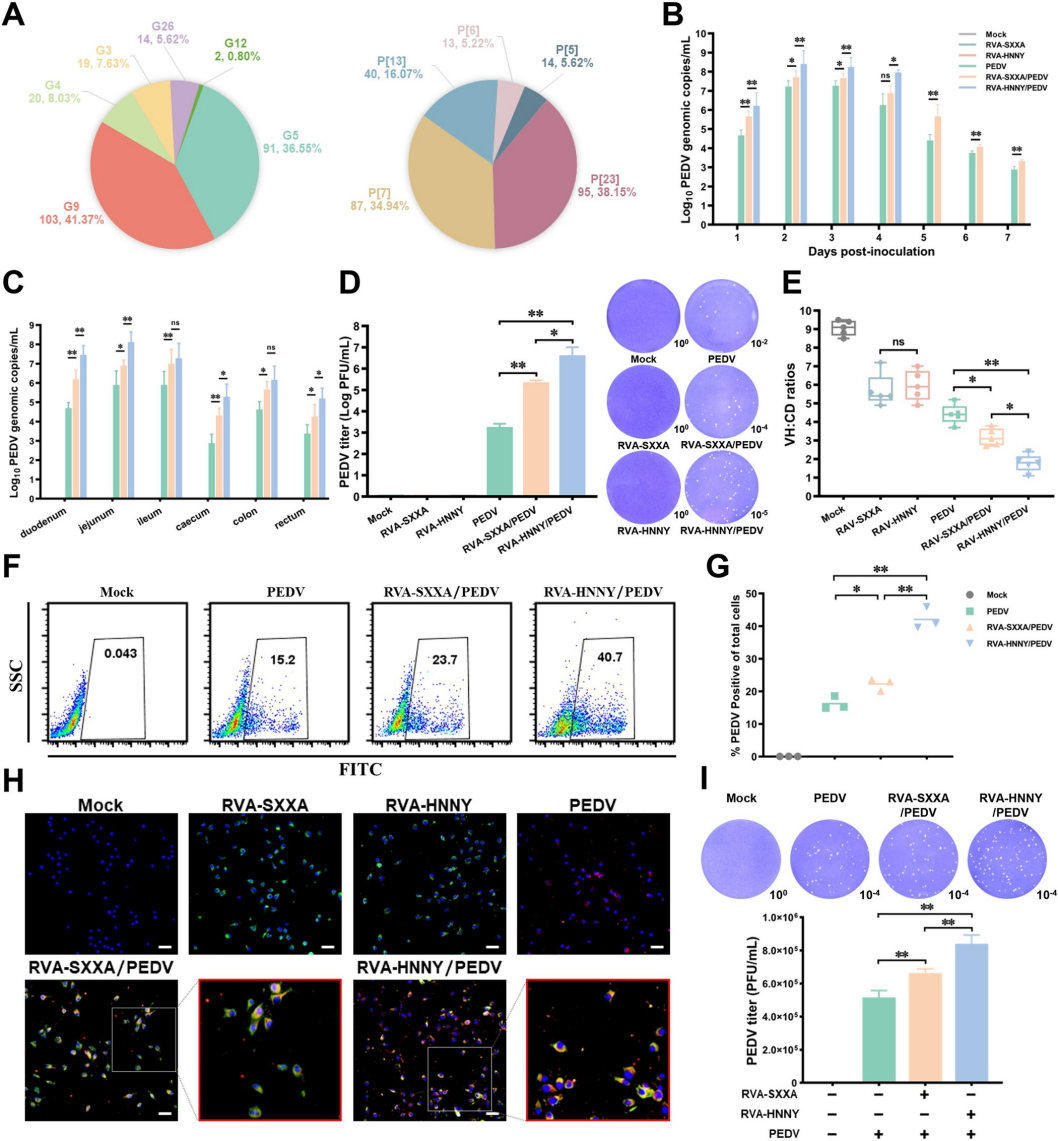
PoRVA infection promotes PEDV replication *in vitro* and *in vivo*. **(A)** Proportions of G-type (VP7) genotypes and P-type (VP4) genotypes among the cases tested positive for PoRVA. **(B)** The number of viral copies of PEDV in fecal swabs determined by quantitative RT–qPCR was monitored after PEDV inoculation for up to 7 days. **(C-E)** Intestinal tissues were collected from piglets 2 days after PEDV infection. The viral load of PEDV in different intestinal segments was quantified by RT–qPCR (C). The virus titer of PEDV in the jejunum of piglets was detected by plaque assay (D). The VH/CD ratios of the jejunum in the different groups were calculated to evaluate the intestinal lesions of the piglets (E). **(F-I)** IPEC-J2 cells were mock-infected or infected with RVA-SXXA or RVA-HNNY (MOI = 1) for 12 h, and then the number of PEDV-positive cells was determined by flow cytometry at 12 h following PEDV infection (MOI = 1) (F). Quantification of the FACS results is shown in the panel. The percentage of PEDV positive cells is presented as the mean ± SD (n = 3) (G). PEDV and PoRVA infection in IPEC-J2 cells was detected by immunofluorescence. The white boxes are magnified at the right of the image. PEDV N (red), PoRVA VP6 (green), DAPI (blue). Scale bar, 100 μm (H). Viral titers of PEDV were detected by plaque assay and quantified (I). * *P* < 0.05, ** *P* < 0.01, ns, not significant.

To further investigate whether PoRVA infection affects the pathogenicity of PEDV, we first analyzed the genotypes of PoRVA in the collected samples. The results showed that the VP7 genotypes G9 and G5 and the VP4 genotypes P[23] and P[7] were the dominant genotypes in clinically PoRVA-infected piglets (Fig 2A). For two isolated representative strains (RVA-SXXA strain and RVA-HNNY strain), phylogenetic analysis based on the nucleotide sequence of VP7 and VP4 revealed that the genotypes of RVA-SXXA and RVA-HMMY were G5P[7] and G9P[23], respectively (S2A Fig). RVA-SXXA and RVA-HMMY showed similar infectivity (S2B–S2E Fig) and replication efficiency in IPEC-J2 cells (S2F Fig), suggesting that there was no significant difference in the in *vitro* characteristics of PoRVA G5P[7] (RVA-SXXA) and PoRVA G9P[23] (RVA-HNNY). Moreover, in the animal infection experiments, RVA-HNNY-infected piglets and RVA-SXXA-infected piglets exhibited similar survival rates (S3A Fig), clinical scores (S3B Fig), fecal scores (S3C Fig), and average weight gain (S3D Fig). Consistently, the PoRVA shedding, tissue viral loading and pathological lesion levels of the intestinal mucosa tissues were not significantly different between RVA-HNNY- and RVA-SXXA-infected piglets, even in the piglets following PEDV challenge (S3E–S3H Fig). However, it should be noted that both RVA-HNNY- and RVA-SXXA-infected piglets showed a lower survival rate (S3A Fig), and more severe clinical signs, as indicated by the clinical score (S3B Fig), fecal score (S3C Fig), and average weight gain (S3D Fig), when challenged with the same doses of PEDV infection than when piglets were challenged with PEDV alone. More notably, compared to RVA-SXXA-infected piglets, RVA-HNNY-infected piglets exhibited a lower survival rate and more severe clinical signs when challenged with the same dose of PEDV (S3A–S3D Fig). These data suggested that PoRVA infection enhances PEDV pathogenicity and that PoRVA G9P[23] (RVA-HNNY strain) enhances PEDV pathogenicity more significantly than PoRVA G5P[7] (RVA-SXXA strain).

To further determine the effects of infection with different genotypes of PoRVA on PEDV infection and pathogenicity, we measured and compared differences in viral shedding, viral loading, and intestinal lesions among groups of piglets. Compared with the piglets in the RVA-SXXA/PEDV group, the piglets in the RVA-HNNY/PEDV group exhibited more PEDV shedding; greater viral loading in the duodenum, jejunum, cecum, and rectum tissues; and more severe intestinal lesions (Figs 2B–2E and S3H). Compared to single PEDV-challenged piglets, both RVA-HNNY/PEDV- and RVA-SXXA/PEDV-infected piglets exhibited more PEDV shedding and greater tissue viral loading (Fig 2B–2E). To determine the effect of different genotypes of PoRVA infection on PEDV replication in cellular experiments, IPEC-J2 cells were mock-infected or infected with RVA-SXXA or RVA-HNNY (MOI = 1) for 12 h and then infected with PEDV (MOI = 1), and PEDV replication levels were assayed 12 h after PEDV infection. Consistent with the *in vivo* observations, both RVA-HNNY and RVA-SXXA infection significantly promoted PEDV replication in IPEC-J2 cells, and RVA-HNNY more significantly promoted PEDV replication in IPEC-J2 cells than did RVA-SXXA (Fig 2F–2I). However, the replication levels of RVA-HNNY and RVA-SXXA in IPEC-J2 cells did not appear to differ significantly when followed by PEDV infection (S3I and S3J Fig). These data collectively demonstrated that PoRVA infection enhances PEDV infection and pathogenicity by promoting PEDV replication, and that PoRVA G9P[23] (RVA-HNNY strain) can more strongly promote PEDV replication to enhance PEDV pathogenicity than can PoRVA G5P[7] (RVA-SXXA strain).

### PoRVA and PEDV infections induce both similarities and differences in cellular metabolic reprogramming

**Fig 3:**
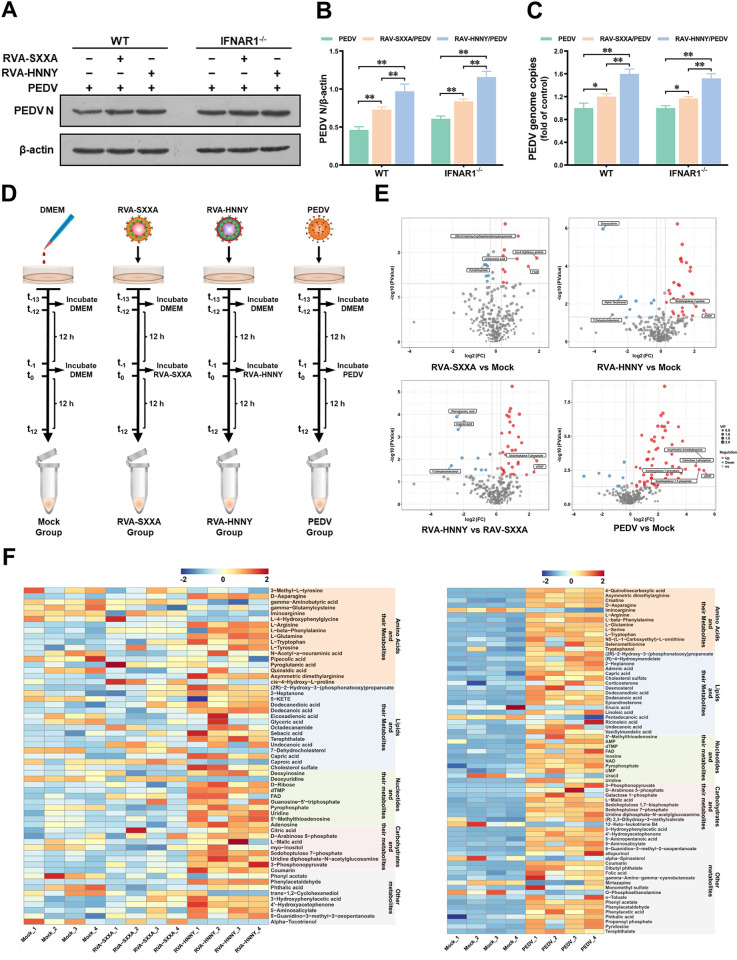
PoRVA infection induces significant changes in cellular metabolites in IPEC-J2 cells. **(A-C)** Wild-type and IFNAR1^-/-^ IPEC-J2 cells were infected with RVA-HNNY or RVA-SXXA at an MOI of 1 for 12 h and then infected with PEDV (MOI = 1) for another 12 h. IPEC-J2 cells were lysed and subjected to western blotting to detect the expression level of the PEDV N protein (A). The proportion of the PEDV N protein was normalized to that of β-actin (B). Cell lysate samples were harvested to quantify PEDV replication via RT–qPCR analysis of the PEDV N gene (C). * *P* < 0.05, ** *P* < 0.01. **(D)** Schematic diagram of the experimental design used for global metabolic profiling. IPEC-J2 cells in the mock, RVA-SXXA, RVA-HNNY, and PEDV groups were first incubated in DMEM for 12 h and then inoculated with DMEM (mock), RVA-SXXA (MOI = 1), RVA-HNNY (MOI = 1), or PEDV (MOI = 1) for another 12 h. Each group included four biological replicates. Metabolites were extracted, and their levels were measured. **(E)** Volcano plots highlighting the metabolites that were increased (red) or decreased (blue) in the RAV-SXXA group, RAV-HNNY group, and PEDV group compared to the mock group. **(F)** Heatmap of differentially abundant metabolites. Each column represents one sample, and each row represents one differentially abundant metabolite. The color keys indicate the levels of different metabolites: red, upregulated; blue, downregulated.

To further investigate how PoRVA infection promotes PEDV replication in IPEC-J2 cells, we tested and compared the effects of RVA-SXXA and RVA-HNNY on PEDV replication at various stages of the viral infection cycle. At the early stage of virus infection, including adsorption and entry, the abundance of PEDV was not affected by PoRVA, but PoRVA significantly increased the viral abundance at the replication stage of PEDV, and the effect of RVA-HNNY was stronger than that of RVA-SXXA (S4 Fig). Since many rotavirus strains have been reported to inhibit Type I interferon induction to regulate a number of viral infections [24], we constructed IFNAR1 knockout IPEC-J2 cells to explore whether PoRVA infection promotes PEDV replication through inhibiting the IFN signaling pathway. However, unexpectedly, PoRVA infection promoted PEDV replication in IFNAR1-deficient (IFNAR1^-/-^) IPEC-J2 cells, and the RVA-HNNY strain promoted PEDV replication more strongly than the RVA-SXXA strain in both IFNAR1^-/-^ cells and wild-type (WT) cells (Fig 3A and 3B). Notably, the increased levels of PEDV replication associated with PoRVA co-infection were similar in IFNAR1^-/-^ and WT IPEC-J2 cells. Additionally, RVA-HNNY strain increased PEDV levels to a greater extent than RVA-SXXA strain in both IFNAR1^-/-^ and WT IPEC-J2 cells (Fig 3C).

Viruses have developed various mechanisms to manipulate host metabolism [15,25]. To test whether PoRVA promotes PEDV replication by remodeling key host metabolic pathways, we performed metabolic profiling to assess the changes in metabolites in RVA-SXXA-infected, RVA-HNNY-infected, and PEDV-infected IPEC-J2 cells (Fig 3D). The results of orthogonal projections to latent structures discriminant analysis (OPLS-DA) showed satisfactory stability and reliability of the UHPLC–QTOF–MS data (S5A Fig). The metabolites were notably altered in abundance after PoRVA infection, and the differentially abundant metabolites are shown in the form of volcano plots (Fig 3E). Compared with those in uninfected IPEC-J2 cells, 11 metabolites were significantly upregulated, and 11 metabolites were significantly downregulated in RVA-SXXA-infected IPEC-J2 cells; in RVA-HNNY-infected IPEC-J2 cells, 32 metabolites were significantly upregulated, and 11 metabolites were significantly downregulated (S5B Fig). The metabolites that were upregulated in both RVA-SXXA- and RVA-HNNY-infected IPEC-J2 cells included glutamine, asparagine, arginine, 4’-hydroxyacetophenone, and dodecanedioic acid. The abundance of these metabolites was greater in RVA-HNNY-infected IPEC-J2 cells than in RVA-SXXA-infected IPEC-J2 cells (Fig 3F and S1 Table). There were differences in the types of metabolites that were downregulated in RVA-SXXA and RVA-HNNY infected IPEC-J2 cells. The metabolites downregulated in RVA-SXXA-infected IPEC-J2 cells included 5-KETE, eicosadienoic acid, glyceric acid, pipecolic acid, and myo-inositol; the metabolites downregulated in RVA-HNNY-infected IPEC-J2 cells included sebacic acid, 7-dehydrocholesterol, alpha-tocotrienol, iminoarginine, and 3-methyl-L-tyrosine. Notably, although the types of differentially expressed metabolites in RVA-SXXA-infected and RVA-HNNY-infected IPEC-J2 cells were not identical, these differentially expressed metabolites were mainly amino acids, lipids, and their derivatives (Fig 3F), which suggests that cellular metabolic perturbations during PoRVA infection are mainly focused on amino acid metabolism and lipid metabolism. This finding is consistent with the results of the KEGG pathway analysis, in which the metabolic pathways altered during PoRVA infection consisted primarily of central carbon metabolism, amino acid biosynthesis, fatty acid biosynthesis, alanine, aspartate, and glutamate metabolism, and arginine biosynthesis (S5C Fig). The above results indicate that PoRVA infection affects lipid and amino acid metabolism, and that there are differences in the types and abundances of differentially expressed metabolites in RVA-SXXA-infected and RVA-HNNY-infected IPEC-J2 cells.

Next, we investigated the similarities and differences in cellular metabolic changes induced by PoRVA infection and PEDV infection. We found that PEDV infection induced more profound reprogramming of cellular metabolism in IPEC-J2 cells (Fig 3F), including 63 significantly upregulated metabolites and 8 significantly downregulated metabolites (S5B Fig). By classifying the PEDV-induced differentially expressed metabolites, we found that similar to PoRVA infection, PEDV infection induced the differential expression of amino acids, lipids and their derivatives (Fig 3F). The metabolites that were upregulated in both PEDV-infected IPEC-J2 cells and PoRVA-infected IPEC-J2 cells included glutamine, arginine, asparagine, dodecanedioic acid, (2R)-2-hydroxy-3-(phosphonatooxy) propanoate, and 4’-Hydroxyacetophenone. There were significant differences in the types of metabolites downregulated in PoRVA-infected and PEDV-infected IPEC-J2 cells. The metabolites downregulated in PEDV-infected IPEC-J2 cells included erucic acid, iminoarginine, pentadecanoic acid, 12-keto-leukotriene B4, mirtazapine, o-phosphoethanolamine, uracil, and α-spinasterol. Overall, the metabolic pathways altered during PEDV infection included mainly amino acid biosynthesis, pyrimidine metabolism, glycine, serine and threonine metabolism, and cofactor biosynthesis (S5C Fig). These results suggest that PEDV infection induces more profound reprogramming of cellular metabolism than does PoRVA infection and that both PEDV and PoRVA infection predominantly affect amino acid metabolism and lipid metabolism in intestinal epithelial cells.

### PoRVA G9P[23] strain infection and PoRVA G5P[7] strain infection have differential effects on PEDV-induced cellular metabolic reprogramming

**Fig 4:**
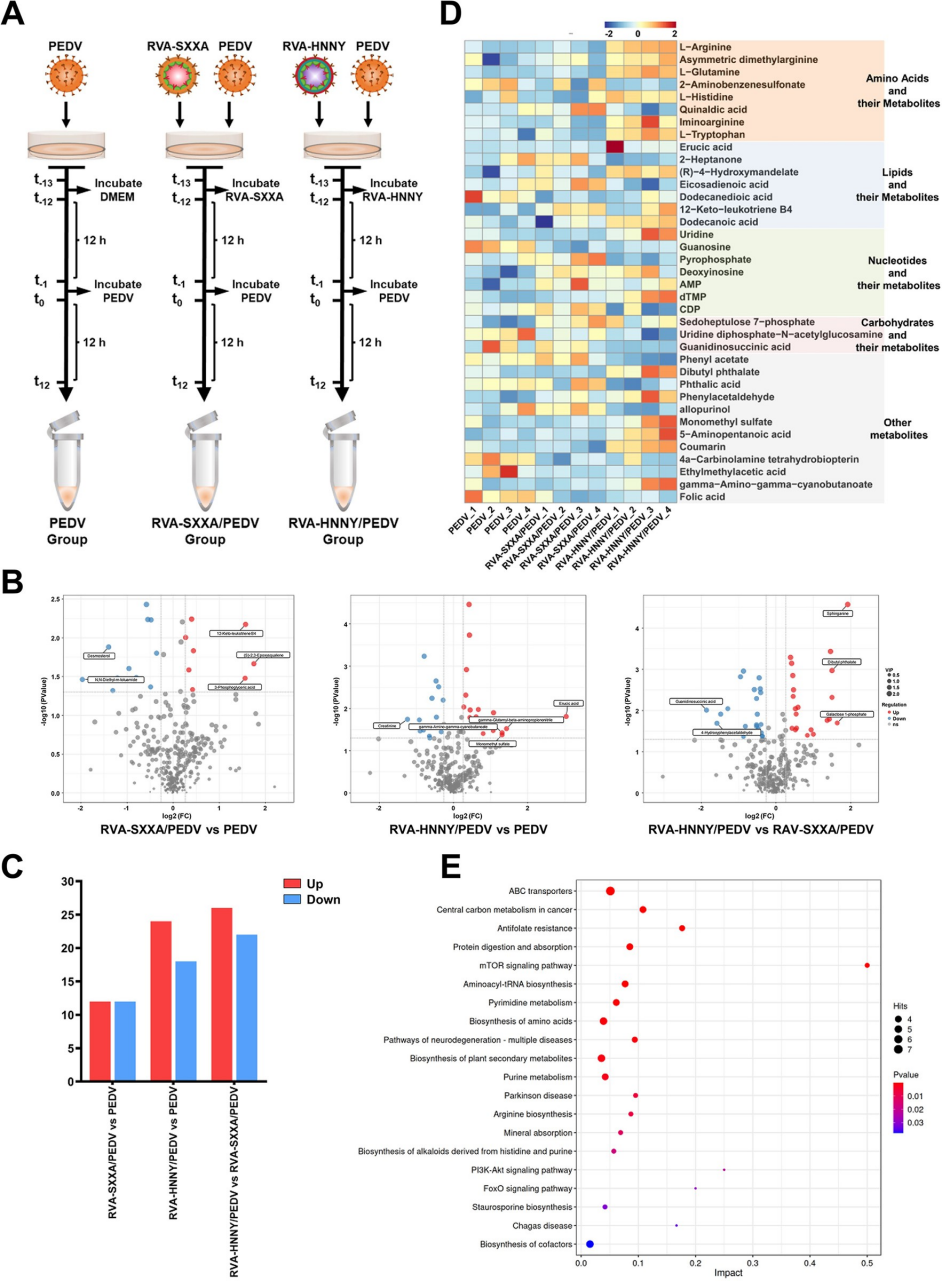
PoRVA G9P[23] strain and PoRVA G5P[7] strain infection differentially affect PEDV-induced cellular metabolic reprogramming. **(A)** Schematic diagram of the global metabolic profiling experimental design. IPEC-J2 cells in the PEDV group were first incubated in DMEM for 12 h and then inoculated with PEDV (MOI = 1) for another 12 h. IPEC-J2 cells in the RVA-SXXA/PEDV group and RVA-HNNY/PEDV group were first infected with RVA-SXXA (MOI = 1) or RVA-HNNY (MOI = 1) for 12 h and then infected with PEDV (MOI = 1) for another 12 h. Each group included four biological replicates. Metabolites were extracted, and their levels were measured. **(B)** Volcano plots highlighting the metabolites that were increased (red) or decreased (blue) among the RAV-SXXA/PEDV, RAV-HNNY/PEDV, and PEDV groups. **(C)** The number of differentially abundant metabolites in different groups. **(D)** Heatmap of differentially abundant metabolites. Each column represents one sample, and each row represents one differentially abundant metabolite. The color keys indicate the levels of different metabolites: red, upregulated; blue, downregulated. **(E)** Bubble plots of the metabolic pathway enrichment analysis of RVA-SXXA/PEDV- and RVA-HNNY/PEDV-infected cells compared to that of PEDV-infected cells. Each bubble in the bubble diagram represents a metabolic pathway. The larger the bubble is, the more metabolites it contains. The x-axis represents a pathway impact value in the topology analysis, and the size is positively correlated with the influence factor. The y-axis represents the metabolic pathways identified in the enrichment analysis.

To obtain a global view of the effects of PoRVA infection on PEDV infection-induced metabolic reprogramming and the differences between the two subtypes of rotaviruses (RVA-HNNY and RVA-SXXA) on the metabolic reprogramming of PEDV-infected cells, we next focused on comparing the changes in metabolites in PEDV-, RVA-SXXA/PEDV-, and RVA-HNNY/PEDV-infected cells (Fig 4A). We found that the RVA-HNNY strain more significantly affected PEDV infection-induced metabolic reprogramming in IPEC-J2 cells than did the RVA-SXXA strain (Fig 4B and 4C). Compared with those in single PEDV-infected cells, 24 metabolites were significantly upregulated, and 18 metabolites were significantly downregulated in cells first infected with RVA-HNNY but subsequently infected with PEDV (Fig 4C). The metabolites with altered abundance mainly included amino acids, nucleotides and their derivatives. The upregulated metabolites included glutamine, arginine, tryptophan, iminoarginine, uridine, CMP, and GMP. The downregulated metabolites included UMP, cytidine, pyrophosphate, and Guanosine (Fig 4D and S1 Table). Compared with those in single PEDV-infected cells, 12 metabolites were significantly upregulated and 12 metabolites were significantly downregulated in cells first infected with RVA-SXXA and subsequently infected with PEDV (Fig 4C). The metabolites with altered abundance mainly included nucleotides and their derivatives. The upregulated metabolites included AMP, UDP, arachidic acid, 3-phosphoglyceric acid, and adenosine. The downregulated metabolites included dodecanedioic acid and guanosine, among others (Fig 4D and S1 Table).

**Fig 5:**
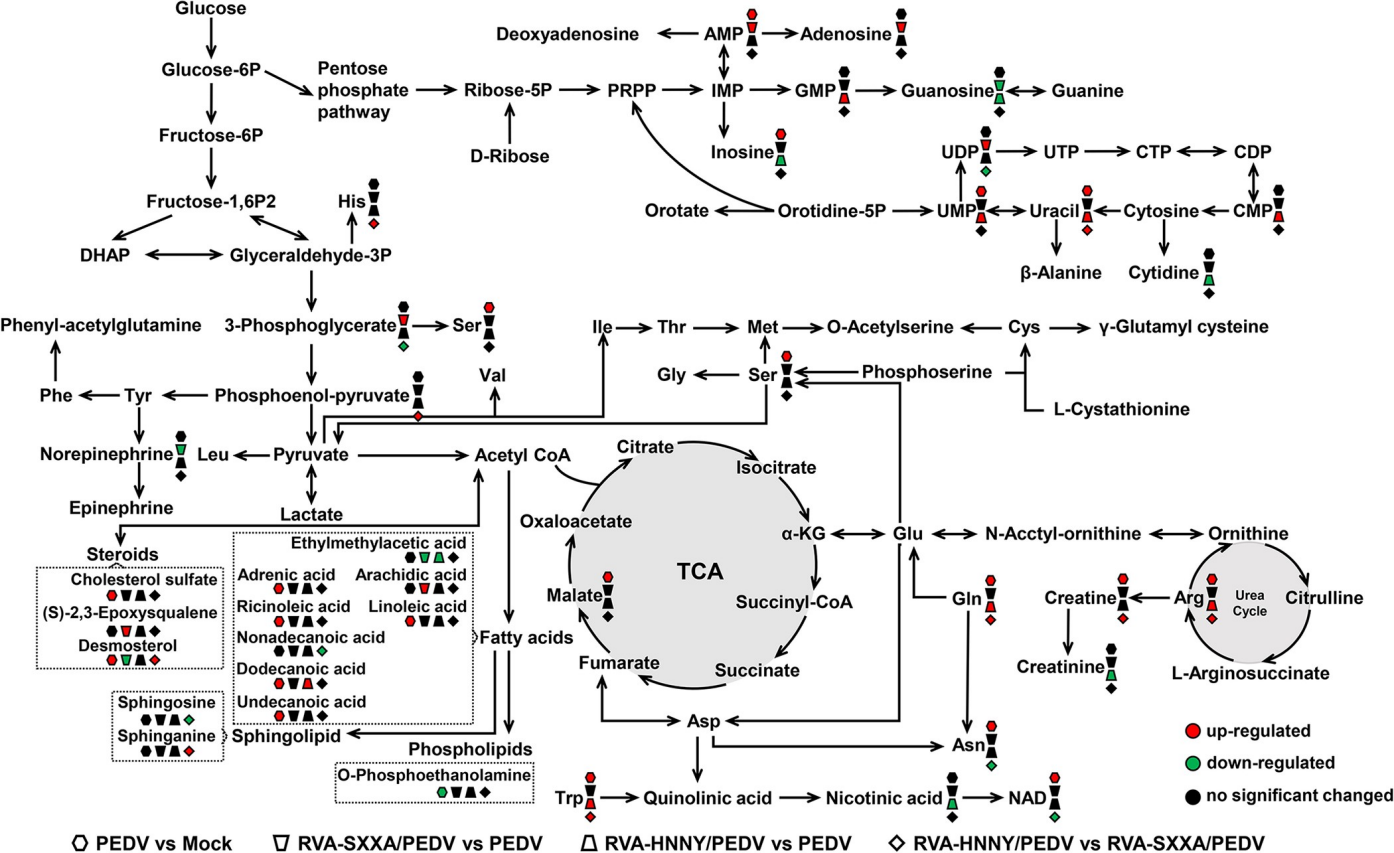
Schematic depicting the significant changes in metabolites and metabolic pathways in untargeted metabolomics. Many metabolites and associated metabolic pathways were significantly altered among the PEDV, RVA-SXXA/PEDV, and RVA-HNNY/PEDV groups. n = 4 samples in each group. VIP >1 and *p* value < 0.05 were used as the screening criteria for significantly differentially abundant metabolites. Red, upregulated; green, downregulated.

Since viruses depend on host cells to obtain the macromolecules and energy needed for their replication, they have evolved numerous strategies to shape cellular metabolism and the biosynthesis machinery [25]. Detailed information on the changes in the abundance of differentially expressed metabolites involved in the dominant metabolic pathways in PEDV-infected cells or in cells first infected with PoRVA and subsequently infected with PEDV was obtained. We found that PEDV infection promoted fatty acid biosynthesis, as evidenced by the accumulation of unsaturated fatty acids, including linoleic acid, ricinoleic acid, and adrenic acid (Fig 5). PEDV infection induces elevated levels of lipid biosynthesis, which facilitates the formation of an enveloped viral replication complex and viral transmission. To our surprise, although our above data indicated that PoRVA infection also induces lipid metabolism reprogramming (Fig 3F), PoRVA infection did not significantly affect the abundance of lipid metabolites during subsequent PEDV infection compared with PEDV infection alone (Figs 4E and 5). The effects of infection with the RVA-SXXA strain or RVA-HNNY strain on the metabolic reprogramming of cells induced by subsequent PEDV infection were mainly focused on amino acid metabolism and nucleotide metabolism (Figs 4D and 5). In particular, RVA-HNNY increased glutamine, arginine, and tryptophan levels more effectively than RVA-SXXA in cells subsequently infected with PEDV (Fig 5). Viral replication was active at 12 h after PEDV infection, and we speculated that the PoRVA-induced changes in metabolite abundance at this time may play an important role in promoting PEDV replication. Whether the differential effects of RVA-HNNY and RVA-SXXA on PEDV infection-induced amino acid metabolism affect PEDV replication remains to be further verified.

### PoRVA G9P[23] promotes PEDV replication more significantly than PoRVA G5P[7], which is related to glutamine utilization

**Fig 6:**
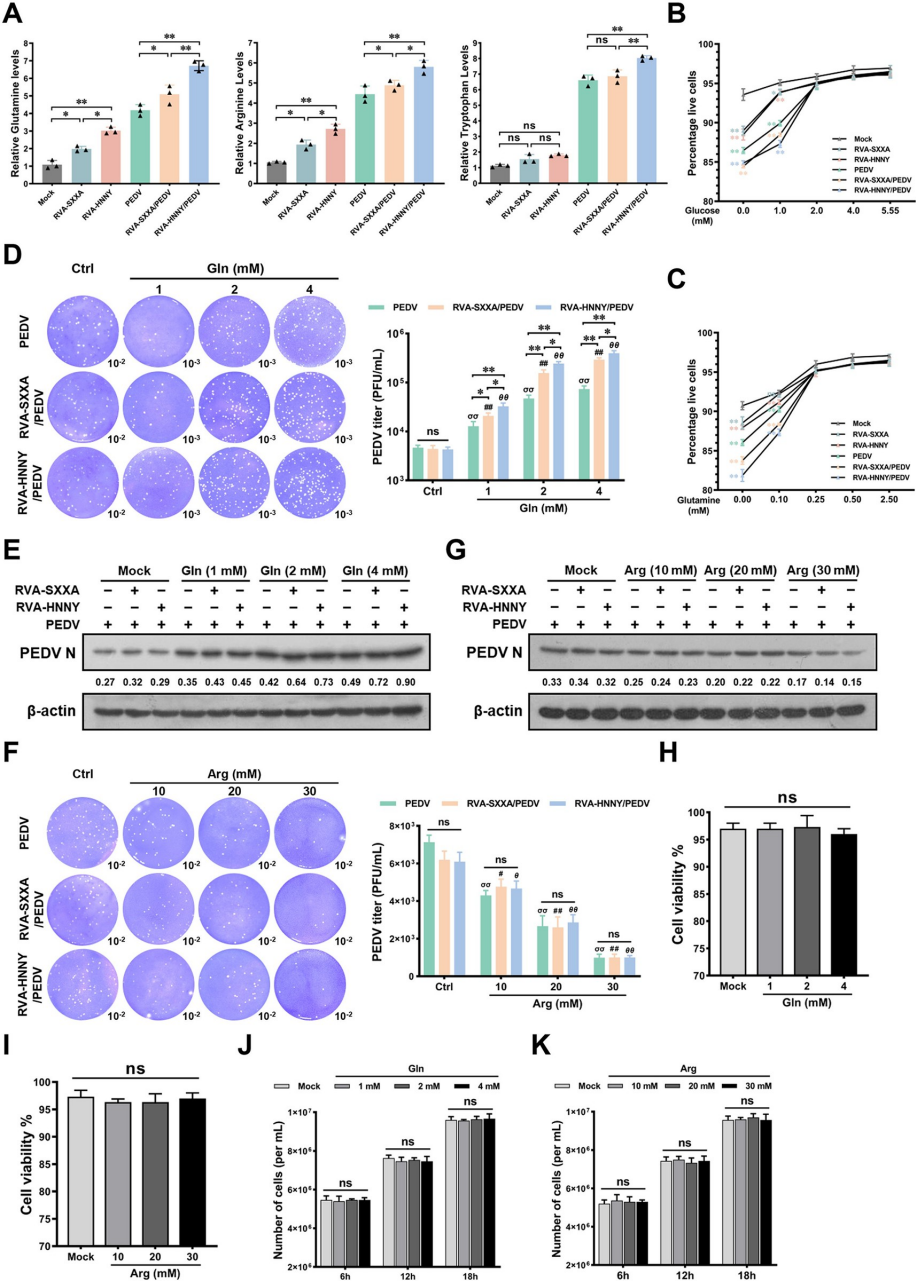
PoRVA promotes PEDV replication by enhancing glutamine uptake. **(A)** Quantification of glutamine, arginine, and tryptophan in mock and infected cells. IPEC-J2 cells in the mock, RVA-SXXA, RVA-HNNY, and PEDV groups were first incubated in DMEM for 12 h and then inoculated with DMEM (mock), RVA-SXXA (MOI = 1), RVA-HNNY (MOI = 1), or PEDV (MOI = 1) for another 12 h. IPEC-J2 cells in the RVA-SXXA/PEDV group and RVA-HNNY/PEDV group were first infected with RVA-SXXA (MOI = 1) or RVA-HNNY (MOI = 1) for 12 h and then infected with PEDV (MOI = 1) for another 12 h. The corresponding metabolite assay kits were used to determine the intracellular concentrations of glutamine, arginine, and tryptophan according to the manufacturer’s protocol. **(B, C)** Viability of IPEC-J2 cells in each group. IPEC-J2 cells in each group were cultured in cell culture media supplemented with various concentrations of exogenous (B) glucose (0.00, 1.00, 2.00, 4.00, or 5.55 mM) or (C) glutamine (0.00, 0.10, 0.25, 0.50, or 2.50 mM). Cell viability assays were performed using a Super-Enhanced Cell Counting Kit-8. **(D-G)** Glutamine is crucial for the ability of PoRVA to promote PEDV replication. IPEC-J2 cells were mock-infected or infected with PoRVA (RAV-SXXA or RAV-HNNY) at an MOI of 1 for 12 h, followed by PEDV (MOI = 1) infection for 12 h. The infected cells were incubated at 37°C in DMEM containing low glucose (2.0 mM) and glutamine (0.25 mM) supplemented with the indicated concentrations of exogenous glutamine (1.0, 2.0, or 4.0 mM) or exogenous arginine (10 mM, 20 mM, or 30 mM). The titers of PEDV in culture media supplemented with exogenous glutamine (D) or arginine (F) were determined via plaque formation assays. PEDV N protein levels in culture media supplemented with exogenous glutamine (E) or arginine (G) were determined by western blotting. **(H-K)** The impact of glutamine and arginine on the viability and number of cells. IPEC-J2 cells were maintained in the presence of glutamine (0, 1, 2, or 4 mM) or arginine (0, 10, 20, or 30 mM) for 6, 12, or 18 h. After 18 h of cultivation, the cytotoxicity of the indicated doses of glutamine (H) and arginine (I) was measured by a CCK-8 assay. Cell growth was detected using a Guava Muse Cell Analyzer (J. K). The error bars represent the SEM (n = 3). ns, not significant; * *P* < 0.05, ** *P* < 0.01; ^*σσ*^ *P* < 0.01 (compared with PEDV group in ctrl treatment); ^***#***^ *P* < 0.05, ^***##***^ *P* < 0.01 (compared with RVA-SXXA/PEDV group in ctrl treatment); ^***θ***^ *P* < 0.05, ^***θθ***^ *P* < 0.01 (compared with RVA-HNNY/PEDV group in ctrl treatment).

Amino acids are substrates for protein synthesis, but they also generate glucose, ATP, and fatty acids and serve as metabolic precursors for numerous biomolecules, including nucleotide bases and signaling molecules [26]. To further analyze which amino acid changes induced by PoRVA affect PEDV replication, the intracellular levels of glutamine, arginine, and tryptophan were measured in each group. We found that compared with control infection, PoRVA (RVA-SXXA and RVA-HNNY) infection significantly upregulated the levels of glutamine and arginine. RVA-HNNY infection induced more significant increases in glutamine and arginine levels than RVA-SXXA infection in cells subsequently infected with PEDV (Fig 6A). Therefore, we next tested whether the more significant promotion of PEDV replication by RVA-HNNY over RVA-SXXA was related to glutamine and arginine. To exclude other factors in the experiment, we initially determined the minimum essential concentrations of glucose (2.0 mM) and glutamine (0.25 mM), which are required for maintaining cell viability during the early stages of viral infection (Fig 6B and 6C). Neither RVA-HNNY nor RVA-SXXA significantly promoted PEDV replication when IPEC-J2 cells were cultured in medium containing the minimum essential levels of glucose and glutamine (Fig 6D and 6E). At minimum essential levels of glucose and glutamine, glutamine supplementation allowed PoRVA to significantly promote PEDV replication in IPEC-J2 cells, and the effect of RVA-HNNY was more pronounced than that of RVA-SXXA (Fig 6D and 6E). In contrast, exogenous supplementation with arginine impaired PEDV replication in a dose-dependent manner (Fig 6F and 6G). Notably, treatment with glutamine or arginine at the indicated concentrations did not result in cellular cytotoxicity (Fig 6H–6K). These results suggest that glutamine plays a key role in the differential promotion of PEDV replication by RVA-HNNY and RVA-SXXA.

### PoRVA G9P[23] infection induces higher levels of SLC1A5 expression than PoRVA G5P[7] infection to enhance glutamine uptake

**Fig 7:**
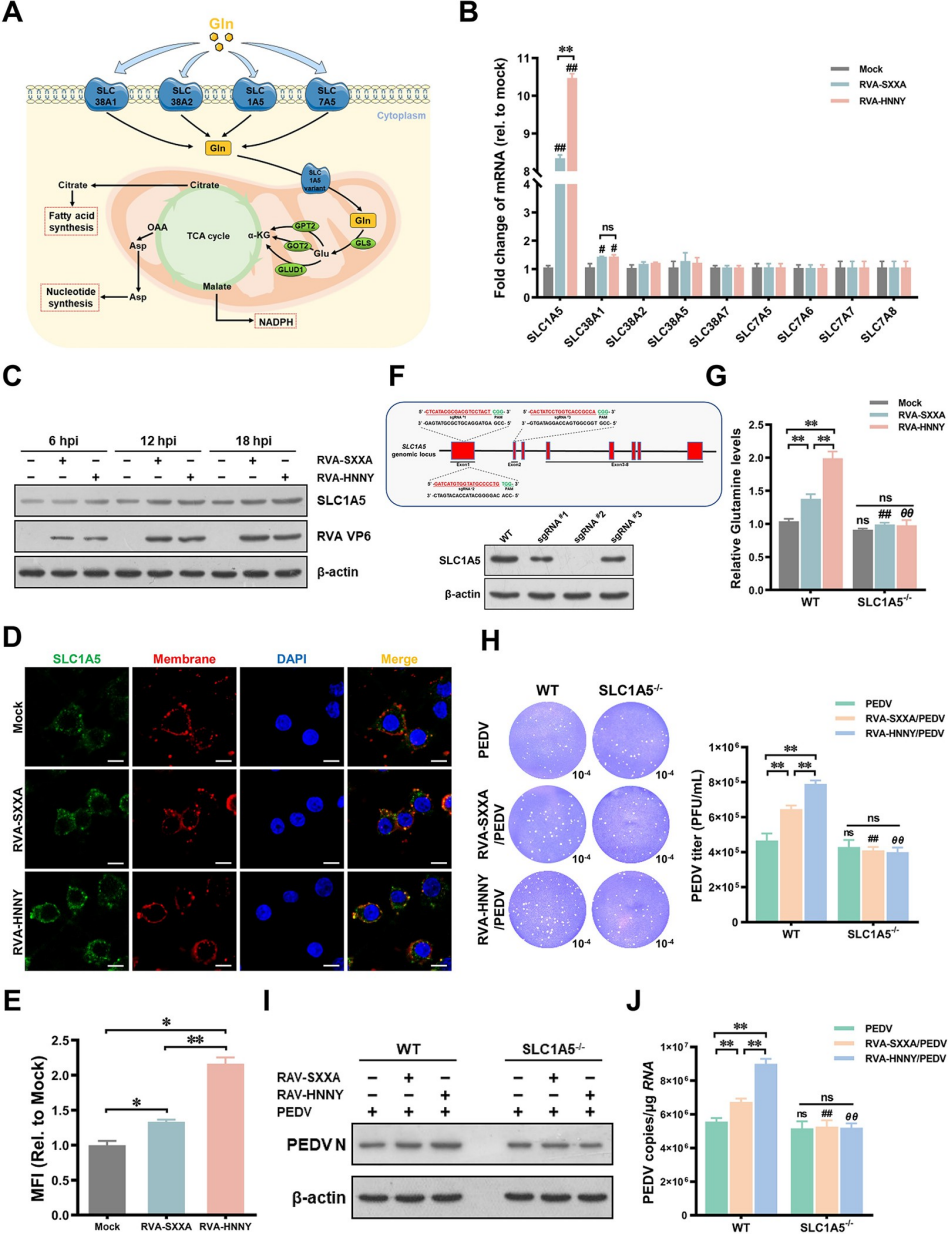
PoRVA infection enhances glutamine uptake by upregulating the expression of SLC1A5. **(A)** Schematic overview of glutamine utilization. **(B)** IPEC-J2 cells were mock-infected or infected with PoRVA (RAV-SXXA or RAV-HNNY) at an MOI of 1 for 6 h. The impact of PoRVA infection on the mRNA expression of the glutamine transporter was determined by RT–qPCR. **(C)** The protein levels of SLC1A5 at different time points after PoRVA infection were detected by western blotting. **(D)** The expression and localization of SLC1A5 were detected by confocal microscopy. IPEC-J2 cells were fixed and stained with an anti-SLC1A5 antibody (green) and DAPI (blue). Moreover, specific probes (red) were used to detect membrane proteins in the cells. **(E)** The fluorescence intensity of the SLC1A5 protein was analyzed by Image J. **(F)** The expression of SLC1A5 in the WT and SLC1A5^-/-^ IPEC-J2 cells were detected by Western blotting. **(G)** Detection of glutamine levels in WT and SLC1A5^-/-^ IPEC-J2 cells after PoRVA infection. WT and SLC1A5^-/-^ IPEC-J2 cells were infected with RVA-SXXA or RVA-HNNY (MOI = 1) for 12 h. The level of glutamine in the cells was detected using a glutamine content assay kit. **(H-J)** WT and SLC1A5^-/-^ IPEC-J2 cells were infected with PoRVA for 12 h, followed by PEDV infection for another 12 h. Afterward, cell lysates were harvested for the detection of viral titers (H), PEDV N protein levels (I), and PEDV viral copies (J). ^***##***^ *P* < 0.01 (compared with RVA-SXXA/PEDV group in ctrl treatment); ^*θθ*^ *P* < 0.01 (compared with the RVA-HNNY/PEDV group in ctrl treatment).

Glutamine is a preferred energy substrate for intestinal epithelial cells and is transported into cells through plasma membrane glutamine transporters (such as SLC1A5, SLC38A1/2, and SLC7A5), which are then used for the biosynthesis of hexosamine, nonessential amino acids (NEAAs), and nucleotides (Fig 7A) [27,28]. To explore the mechanism by which PoRVA infection promotes an increase in glutamine, the mRNA levels of glutamine transporters in PoRVA-infected IPEC-J2 cells were detected to screen for glutamine transporters involved in glutamine uptake during RVA-HNNY or RVA-SXXA infection. PoRVA infection increased the mRNA levels of SLC1A5 and SLC38A1, but did not affect the mRNA levels of other glutamine transporters (Fig 7B). We harvested cells at 6, 12, and 18 h after PoRVA infection (RVA-HNNY and RVA-SXXA) to further investigate the expression of SLC1A5 during PoRVA infection. The results showed that SLC1A5 expression was time-dependently upregulated in PoRVA-infected cells, and RVA-HNNY induced a more pronounced upregulation of SLC1A5 at both the mRNA and protein levels than RVA-SXXA (Fig 7C). The results of the immunofluorescence experiment also demonstrated that the expression of SLC1A5, which is localized on the cell membrane, was greater in RVA-HNNY-infected cells than in RVA-SXXA-infected cells (Fig 7D and 7E).

To investigate the influence of SLC1A5 on glutamine uptake during PoRVA infection, we constructed SLC1A5 knockout IPEC-J2 cells for PoRVA infection (Fig 7F). In SLC1A5^-/-^ IPEC-J2 cells, PoRVA infection did not increase the intracellular glutamine concentration (Fig 7G). As expected, neither RVA-HNNY infection nor RVA-SXXA infection was effectively increased the PEDV titer or viral yield in SLC1A5^-/-^ IPEC-J2 cells (Fig 7H–7J). Collectively, these results suggest that PoRVA G9P[23] infection leads to a more significant increase in SLC1A5 expression than G5P[7] infection, which is critical for the uptake of glutamine and the promotion of PEDV infection.

### PoRVA G9P[23] infection induces glutamine anaplerosis into the TCA cycle more efficiently than PoRVA G5P[7] infection, which is beneficial for PEDV replication

**Fig 8:**
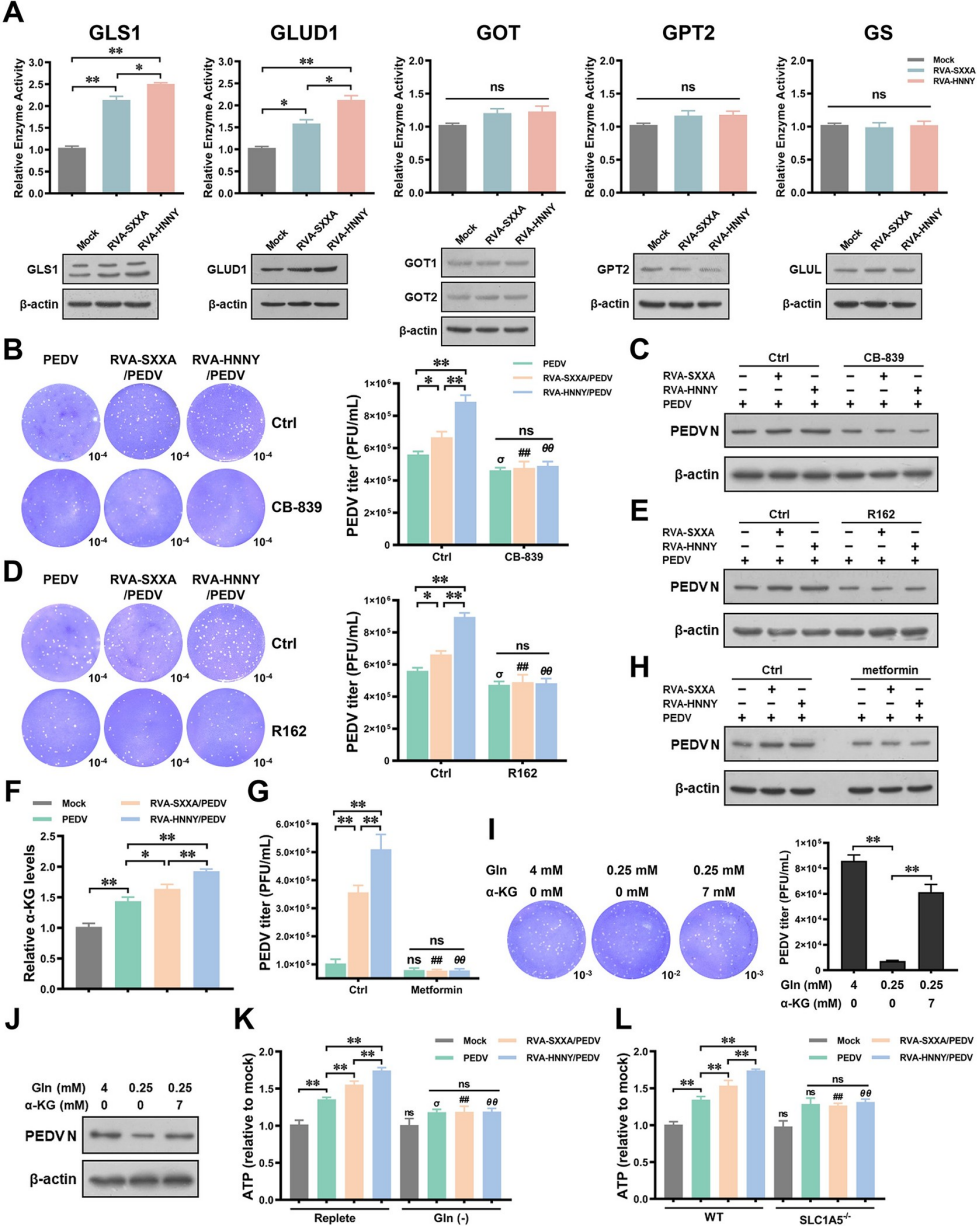
PoRVA G9P[23] infection induces glutamine anaplerosis into the TCA cycle more efficiently than PoRVA G5P[7] infection, which is beneficial for PEDV replication. **(A)** IPEC-J2 cells were infected with RVA-SXXA or RVA-HNNY (MOI = 1) for 12 h. The activity and expression of multiple enzymes involved in glutaminolysis were determined by a corresponding activity assay kit and western blot. **(B-E)** IPEC-J2 cells were mock-infected or infected with RVA-SXXA or RVA-HNNY (MOI = 1) for 12 h, followed by PEDV (MOI = 1) infection for 12 h. Vehicle (DMSO), CB839 (10 μM), or R162 (20 μM) was added 4 h after PoRVA infection of IPEC-J2 cells. The titers of PEDV were determined using a plaque formation assay (B, D). PEDV N protein expression was determined by western blot (C, E). **(F)** IPEC-J2 cells were cultured in DMEM supplemented with low glucose (2.0 mM) and glutamine (4 mM). IPEC-J2 cells were mock-infected or infected with RVA-SXXA or RVA-HNNY (MOI = 1) for 12 h, followed by PEDV (MOI = 1) infection for another 12 h. The titers of PEDV were determined using a plaque formation assay. The intracellular concentration of α-KG in mock and infected cells was determined by an α-KG content assay kit. **(G, H)** Metformin treatment suppressed the promotion of PEDV infection by PoRVA. IPEC-J2 cells were cultured with an adequate concentration of glutamine (4.0 mM) and a minimum concentration of glucose (2.0 mM). IPEC-J2 cells were mock-infected or infected with RVA-SXXA or RVA-HNNY (MOI = 1) for 12 h, followed by infection with PEDV (MOI = 1) for another 12 h in the presence of metformin (5 mM). PEDV titers were determined using a plaque formation assay (G). PEDV N protein expression was determined by western blot (H). **(I, J)** α-KG rescues PEDV replication under low-glutamine conditions. PEDV-infected IPEC-J2 cells were cultured in adequate glutamine medium (4 mM glutamine, 2.0 mM glucose) or low glutamine (0.25 mM) supplemented with or without α-KG (7 mM). IPEC-J2 cells were infected with PEDV (MOI of 1) for 12 h; PEDV titers were determined using a plaque formation assay (I). PEDV N protein expression was determined by western blot (J). **(K)** IPEC-J2 cells were mock-infected or infected with RVA-SXXA or RVA-HNNY at an MOI of 1 for 12 h, followed by infection with PEDV (MOI = 1) for another 12 h. IPEC-J2 cells were cultured in complete medium or glutamine-free medium after PoRVA infection. The levels of intracellular ATP were detected using an ATP content assay kit at 12 hours after PEDV infection. **(L)** WT or SLC1A5^-/-^ IPEC-J2 cells were mock-infected or infected with RVA-SXXA or RVA-HNNY at an MOI of 1 for 12 h followed by infection with PEDV (MOI = 1) for another 12 h. The levels of intracellular ATP were detected using an ATP content assay kit at 12 h after PEDV infection. Error bars are standard deviations of the means for 3 separate determinations. ^*σ*^ *P* < 0.05, (compared with the PEDV group in ctrl treatment group); ^***##***^*P* < 0.01 (compared with the RVA-SXXA/PEDV group in ctrl treatment group); ^***θθ***^ *P* < 0.01 (compared with the RVA-HNNY/PEDV group in ctrl treatment group).

Glutamine plays a versatile role in cellular metabolism. In particular, it serves as a precursor for nucleotides, fatty acids, and other NEAAs, and as a substrate for oxidative phosphorylation. To further investigate the changes in glutamine metabolic pathways during PoRVA infection, we measured the enzymatic activities and expression of various metabolic enzymes involved in glutaminolysis at 12 h after PoRVA infection (RVA-HNNY and RVA-SXXA). Among these genes, glutaminase 1 (GLS1) and glutamate dehydrogenase 1 (GLUD1) enzyme activity and protein expression were elevated, while glutamic oxaloacetic transaminase (GOT1 and GOT2), glutamate pyruvate transaminase 2 (GPT2), and glutamine synthase (GS) were unchanged during PoRVA infection (Fig 8A). To verify the importance of glutaminolysis in PEDV replication, the transcript levels of GLS1 and GLUD1 were silenced in IPEC-J2 cells with siRNAs. The knockdown efficiencies of the siRNAs were verified at 24 h post transfection (S6A Fig). The GLS1 and GLUD1 transcript levels were reduced by 50% and 70%, respectively, in comparison to those in the control siRNA group (S6B Fig). PoRVA was unable to promote PEDV infection in siGLS1 cells; the promotion of PEDV replication by PoRVA was significantly impaired in siGLUD1 cells compared to si-NC cells (S6C–S6E Fig). We then tested whether pharmacological small molecule inhibitors of GLS1 and GLUD1 affect the ability of PoRVA to promote PEDV replication. The GLS inhibitor CB-839 is known to block the catalytic center of GLS [29]. The GLUD1 inhibitor R162 binds directly to GLUD1 and inhibits its activity, resulting in decreased intracellular fumarate levels and increased ROS levels [30]. Both inhibitors suppressed the ability of PoRVA to promote PEDV replication (Fig 8B–8E).

We hypothesized that glutamine anaplerosis into the TCA cycle provides biosynthetic precursors and sustains ATP production, thereby facilitating PEDV replication. The level of α-ketoglutarate (α-KG) was significantly greater in cells infected with RVA-HNNY or RVA-SXXA followed by PEDV than in cells infected with PEDV alone. The levels of α-KG were greater in cells infected with RVA-HNNY followed by PEDV than in cells infected with RVA-SXXA followed by PEDV (Fig 8F). These results suggest that PoRVA infection induces anaplerotic reactions that convert glutamine into TCA cycle intermediates. To further examine the role of the glutamine-dependent TCA cycle in the process by which PoRVA promotes PEDV replication, IPEC-J2 cells were cultured with an adequate concentration of glutamine (4.0 mM) and a minimum concentration of glucose (2.0 mM). Metformin, which suppresses the TCA cycle and elevates lactate production [31], was used to inhibit the TCA cycle in subsequent experiments. Our results showed that treatment with a nontoxic concentration of metformin (5 mM) significantly suppressed the ability of PoRVA to promote PEDV replication (Figs 8G, 8H, and S6E), suggesting that the TCA cycle is involved in PEDV replication. The addition of α-KG to low-glutamine medium (0.25 mM glutamine, 2.0 mM glucose) partially rescued PEDV replication (Fig 8I and 8J), suggesting that glutamine can be converted to α-KG as a TCA cycle intermediate to support PEDV replication.

**Fig 9:**
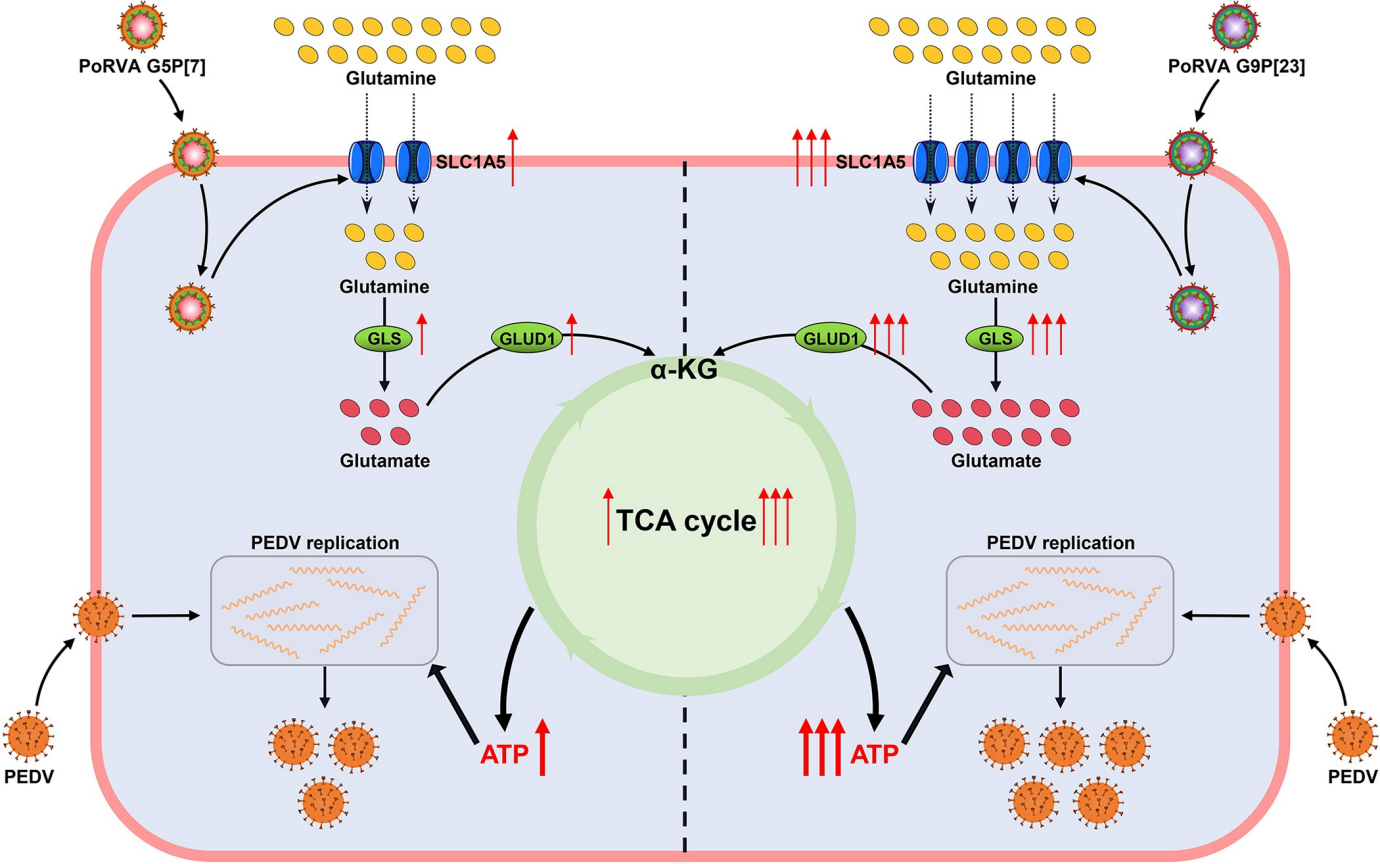
Schematic illustration of the mechanisms by which PoRVA G5P[7] and G9P[23] promote PEDV replication. The expression of SLC1A5, a glutamine transporter, was upregulated during infection with the two subtypes of PoRVA. Compared to that of PoRVA G5P[7] (RVA-SXXA strain), the expression of SLC1A5 was more significantly upregulated during PoRVA G9P[23] (RVA-SXXA strain) infection, which greatly increased glutamine uptake and resulted in increased intracellular glutamine levels. Intracellular glutamine is hydrolyzed to glutamate by glutaminase 1 (GLS1), and glutamate is then converted to α-KG by dehydrogenase 1 (GLUD1), further entering the TCA cycle to increase ATP production. Notably, the metabolic enzyme activity and expression levels of GLS1 and GLUD1 were more markedly upregulated in cells infected with PoRVA G9P[23]. Thus, PoRVA G9P[23] infection remarkably contributes to more ATP synthesis to support PEDV replication than does PoRVA G5P[7] infection.

Viruses rely on cellular energy metabolism to support virion replication and assembly [32]. We found that PoRVA increased the concentration of ATP in cells and that RVA-HNNY had a stronger effect than RVA-SXXA on ATP production (Fig 8K). The increase in the cellular ATP content induced by PEDV infection was more pronounced when the cells were first infected with PoRVA (Fig 8K). However, PoRVA infection was not effective at increasing cellular ATP levels under culture conditions lacking glutamine in the medium. Moreover, glutamine deprivation inhibited the ability of PoRVA infection to promote the increase in cellular ATP levels induced by secondary PEDV infection (Fig 8K), as well as the ability of PoRVA to promote PEDV replication (Fig 6E). In SLC1A5^-/-^ IPEC-J2 cells, the effect of PoRVA infection on the subsequent PEDV infection-induced increase in cellular ATP levels disappeared (Fig 8L). These results suggest that PoRVA induces glutaminolysis and glutamine entry into cells via SLC1A5 and increases the anaplerotic flux of glutamine into the TCA cycle and ATP production, which promotes PEDV replication. In addition, RVA-HNNY infection induces glutamine anaplerosis into the TCA cycle more efficiently than RVA-SXXA infection, which is beneficial for PEDV replication (Fig 9).

## Discussion

Previous studies have reported the simultaneous detection of multiple porcine enteric viruses in the intestinal tract and feces [33], and the coexistence of multiple enteric viruses may have synergistic or antagonistic effects on viral infections [11,34]. PoRVA and PEDV are two widespread enteric viruses that cause acute viral gastroenteritis in pigs and wild boars worldwide, resulting in significant economic losses to the pig rearing industry [12,35,36]. PoRVA and PEDV coinfection is fairly common in natural infections, and leads to increased mortality among newborn piglets [37]. However, the current epidemiological status of PoRVA and PEDV in China is still poorly understood. In the present study, we analyzed 582 diarrhea samples from four provinces (Shaanxi, Gansu, Shanxi, and Henan) in Midwest China between 2020 and 2022 to investigate the spread of porcine enteric viruses in pig herds in this region. The results of the epidemiological investigation of enteric viruses revealed that the main causative agents of diarrhea in pig herds were PoRVA (54.81%, 319/582) and PEDV (42.44%, 247/582). More importantly, we found that a large number of pigs with diarrhea (210/582) were infected with both PoRVA and PEDV, and we demonstrated a potential connection between infection with these two viruses (S1D Fig). Therefore, we hypothesized that the pathogenic potential of PoRVA-PEDV coinfection in the field was significantly higher than that caused by PoRVA or PEDV alone, because PoRVA infection promoted secondary PEDV infection. We verified in animal experiments the hypothesis that PoRVA infection promotes the pathogenicity and mortality of PEDV infection. Furthermore, we found that PoRVA infection promoted PEDV infection by increasing PEDV replication *in vivo* and *in vitro*. However, inconsistent with natural infection, there were no significant changes in clinical signs and mortality in PKV/PEDV-infected piglets compared to PEDV-infected piglets. This difference might be due to a variety of factors; antiviral immunity of infected-pigs, coinfection with other pathogens, the rearing environment, and the stress response in swine, as well as other factors that might influence mortality in naturally infected pigs.

PoRVA was the main cause of acute diarrhea in piglets among all of the RV groups. PoRVA strains are antigenically heterogeneous, and are classified into multiple G and P types defined by the outer capsid proteins VP7 and VP4 [38]. Consistent with a previous study [39], our study suggested that multiple genotype combinations of PoRVA are circulating in pig herds in Midwest China, with G9P[23] and G5P[7] predominating. We isolated the RVA-HNNY and RVA-SXXA strains from diarrhea samples as representative strains of the G9P[23] and G5P[7] genotypes, respectively. Interestingly, although both RVA-HNNY (G9P[23]) and RVA-SXXA (G5P[7]) were able to promote PEDV replication, RVA-HNNY infection promoted PEDV replication more significantly than RVA-SXXA infection. This phenomenon is associated with the ability of these two subtypes of rotaviruses to increase viral abundance during the replication phase of PEDV infection and is not dependent on the IFN-I signaling pathway. Our data revealed that PoRVA and PEDV coinfection appeared obvious synergistic pathogenic effects. In coinfection models of swine viruses, many reports have indicated that virus replication is affected during coinfection [40]. We also found that PoRVA promoted PEDV replication in piglets coinfected with these two viruses and that, compared with PoRVA G5P[7], G9P[23] increased PEDV replication more significantly. There were no significant differences in the genome replication or encoded protein expression of PoRVA between piglets in the PoRVA/PEDV groups and piglets infected with different genotypes of PoRVA. However, our findings do not imply that PEDV has no influence on PoRVA during coinfection with these two viruses. The effect of PEDV on the viral infectivity of PoRVA remains unclear and requires further study in future.

Viruses have evolved to manipulate host cellular metabolism to support the energetic and biosynthetic requirements for optimal replication [41]. We analyzed the metabolomic profiles of PoRVA-infected IPEC-J2 cells using a global metabolomic screen. PoRVA infection predominantly affected amino acid metabolism and lipid metabolism, and there were differences in the types and abundances of differentially expressed metabolites between RVA-SXA-infected and RVA-HNNY-infected cells. Compared to PoRVA infection, PEDV infection induced more profound metabolic reprogramming in IPEC-J2 cells (Fig 3). Like in PoRVA infection, PEDV infection also regulated cellular amino acid metabolism. Amino acid metabolism plays an important role in maintaining cellular homoeostasis [26]. Further analysis of the metabolomic results revealed that glutamine levels were increased in both PoRVA-infected and PEDV-infected cells.

Glutamine, as a source of both energy and carbon and nitrogen backbones, is the major respiratory fuel for enterocytes and immune cells [42]. Glutamine is a nonessential amino acid (NEAA), but in the case of viral infections, the cellular demand for glutamine far exceeds the rate of its own synthesis, making glutamine an essential amino acid for maintaining normal cellular conditions. Therefore, glutamine is also considered a conditionally essential amino acid [28,42]. Glutamine may provide carbon or nitrogen sources for the synthesis of viral proteins and nucleotides, which is necessary for viral replication. Recent studies have demonstrated that viruses may increase glutamine uptake and utilization for viral replication. For instance, hepatitis C virus (HCV) infection increases glutamine utilization and dependence, and inhibiting glutamine metabolism attenuates HCV infection and oxidative stress [43]. Glutamine is required for (red-spotted grouper nervous necrosis virus) RGNNV replication; glutamine deficiency does not affect cell viability but dramatically inhibits RGNNV replication [44]. Similarly, adenovirus-induced reprogramming of glutamine metabolism through MYC activation promotes optimal progeny virion generation [45]. Our data suggest that RVA-HNNY induces glutamine uptake more significantly than RVA-SXXA, which promotes PEDV replication. We examined the expression of glutamine transporter proteins in PoRVA-infected intestinal epithelial cells and determined that PoRVA infection increases glutamine uptake primarily through the upregulation of SLC1A5/ASCT2 expression. Glutamine transporters are membrane-bound proteins that mediate the transfer of glutamine into and out of cells [46]. Among these genes, SLC38A1/2/5/7, SLC7A5/6/7/8, and SLC1A5 have been shown to be expressed in intestinal epithelial cells [47]. The knockdown of SLC1A5 not only inhibited the increase in intracellular glutamine levels induced by PoRVA infection, but also eliminated the effect of PoRVA infection on PEDV replication. This finding reveals the importance of SLC1A5 in regulating glutamine uptake during PoRVA infection. Similarly, HCV infection upregulates SLC1A5 to drive glutamine addiction in cancer cells [43], and a recent study demonstrated that SLC1A5 membrane localization increases the presence of hACE2 receptors able to bind the SARS-CoV-2-RBD [48]. Our data, as well as recent studies on SLC1A5 in virus-infected cells, suggest that SLC1A5 plays a significant role in the virus life cycle.

Glutamine is involved in several important metabolic processes in proliferating cells, where it is degraded by glutaminase to glutamate, which is further metabolized to metabolic intermediates to participate in different anabolic and catabolic pathways [49]. Tumor cells are known to exhibit metabolic reliance on glutamine and can compensate for the loss of glucose-derived TCA cycle intermediates by activating glutaminolysis [50]. Several viral infections also induce glutaminolysis to increase the anaplerotic flux of glutamine into the TCA cycle and replenish TCA cycle intermediates in infected cells, a process that supports bioenergetics and biosynthesis for optimal virus replication [43,45]. Here, we observed that PoRVA infection increased the enzymatic activity and expression of GLS1 and GLUD1, which are related to glutamine catabolism, with RVA-HNNY infection having a more pronounced effect than RVA-SXXA infection. However, there were no significant changes in the activity or expression levels of glutamine synthetase (GS), which is responsible for catalyzing the conversion of glutamate to glutamine. This finding suggested that the increase in the intracellular glutamine content induced by PoRVA infection was due to glutamine uptake rather than glutamate transformation. Both CB839 (an inhibitor of GLS) and R162 (an inhibitor of GLUD1) suppressed the ability of PoRVA to promote PEDV replication, suggesting that glutamine catabolism is critical for the promotion of PEDV replication by PoRVA infection. We further demonstrated that PoRVA infection induces glutamine to enter the TCA cycle via α-KG, which is involved in PEDV replication (Fig 8F–8H). The deleterious effects of glutamine withdrawal on PEDV replication could be partially rescued by α-KG. We did not observe a complete recovery of PEDV viral yield by α-KG, probably due to the requirement for glutamine to support additional metabolic pathways during PoRVA infection such as the synthesis of nucleotides and other amino acids for viral replication and the generation of progeny viruses. Compared with RVA-SXXA infection, RVA-HNNY infection more significantly increased the intracellular ATP content, which provided more energy for PEDV replication in secondary PEDV infections. Further studies on whether PoRVA infection-induced glutamine metabolism promotes PEDV replication through other pathways will help to elucidate the complicated roles of virus-induced metabolic reprogramming.

In summary, we found that coinfections of PoRVA and PEDV, characterized by high mortality, are extremely common in pig herds in Midwest China. PoRVA infection promotes PEDV infection *in vivo* and *in vitro*, and PoRVA G9P[27] (RAV-HNNY strain) enhances PEDV replication more significantly than PoRVA G5P[7] (RAV-SXXA strain). Our results showed that PoRVA infection distinctly altered glutamine metabolism in porcine small intestinal epithelial cells and that, compared with RVA-SXXA, RAV-HNNY induced a more significant increase in the intracellular glutamine content in cells. Elevated intracellular glutamine promoted PEDV replication. Then, we identified the glutamine metabolic pathways that promote PEDV replication. Taken together, our findings provide a better understanding of how PoRVA infection induces metabolic reprogramming to promote PEDV replication and the reasons for the differences in the ability of PoRVA subtypes to promote PEDV replication.

## Materials and methods

### Ethics statement

The use of animals was conducted under the guidelines of Animal Ethics Procedures and Guidelines of the People’s Republic of China and were approved by the Institutional Animal Care and Use Committee (IACUC) of Northwest A&F University (permit number: XN2023-1101; onset and demise dates: March 2, 2023 to March 15, 2023). The collection of clinical samples in this study were all carried out from nonprotected areas and approved by the farm owners.

### Sample collection and PCR detection of swine enteric viruses

In this study, a total of 582 diarrhea samples from 34 pig farms were collected in Shaanxi, Gansu, Shanxi, and Henan provinces in Midwest China between October 2020 and December 2022. Basic information about the farms, including farm size and history of enterovirus infection, was recorded. Farms numbered 1, 2, 6, 14, 15, 17, 20, 21, 26, 28, 29, and 32 had a history of enteric virus infections. Among 582 diarrhea samples, 170 samples (87 intestine tissues and 83 feces) were from 11 pig farms in Shaanxi Province, 143 samples (76 intestine tissues and 67 feces) were from 8 pig farms in Gansu Province, 138 samples (67 intestine tissues and 71 feces) were from 8 pig farms in Henan Province, and 131 samples (73 intestine tissues and 58 feces) were from 7 pig farms in Shanxi Province. The intestinal homogenates and fecal samples were diluted with phosphate-buffered saline (PBS) to 10% (wt/vol) suspensions, and then the supernatants were collected by centrifugation at 5000 × g for 15 min at 4°C. The clarified supernatants were stored at -80°C until viral detection.

Total RNA from intestinal tissues and stool samples was extracted using TRIzol reagent (Invitrogen, 15596026), and cDNA was reverse transcribed using the reverse transcriptase (TaKaRa, 639537) according to the manufacturer’s instructions. DNA extraction from intestinal tissues and stool samples was performed according to the protocol previously described by Wang et al. [51]. Virus-specific RT-PCR assays were used to detect PEDV, TGEV, PoRVA, swine acute diarrhea syndrome coronavirus (SADS-CoV), porcine kobuvirus (PKV), porcine sapelovirus (PSV), porcine deltacoronavirus (PDCoV) and porcine bocavirus (PBoV). The specific sequences of primers used for various pathogens are summarized in S1 Table.

### Cell lines and viruses

Porcine kidney (PK-15) cells were stocks in our lab. Porcine intestinal epithelial (IPEC-J2) cells and African green monkey kidney (MA104) cells were kind gifted d by Prof. Guangxing Li (Northeast Agricultural University, China). African green monkey kidney (Vero) cells (ATCC, CCL-81) were obtained from the American Type Culture Collection (ATCC). IPEC-J2, Vero and PK-15 were cultured in Dulbecco’s modified Eagle medium (DMEM/F-12) (Gibco, 11330032) supplemented with 10% fetal bovine serum (FBS, HyClone, SH30084.03), 100 U/mL penicillin, and 10 μg/mL streptomycin. MA104 were cultured in M199 medium (Gibco, 12340030) supplemented with 10% FBS, 100 U/mL penicillin, and 10 μg/mL streptomycin. All of the cell lines were maintained at 37°C and 5% CO_2_ in a humidified atmosphere.

The isolation of RVA-SXXA (genotype G5P[7]; GenBank: OR091159) and RVA-HNNY (genotype G9P[23]; GenBank: OR091162) on MA104 cells in the presence of 5 μg/mL trypsin as described previously [52]. PKV CH/SX (GenBank: OR778375) isolation was conducted on PK-15 cells as previously described [53]. PEDV SXXY-1 (GenBank: OR472492) isolation was conducted on Vero cells in the presence of 5 μg/mL trypsin as our previous study [54].

### Reagents and antibodies

Glutamine (Gln) content assay kit (BC5305), arginine(Arg)content assay kit (BC5635), glutaminase (GLS) assay kit (BC1455), dehydrogenase (GDH) assay kit (BC1465), glutamic-oxalacetic transaminase(GOT)assay kit (BC1565), glutamic-pyruvic transaminase (GPT) activity assay kit (BC1555), glutamine synthetase (GS) activity assay kit (BC0915), α-ketoglutaric acid (α-KG) content assay kit (BC5425), and ATP content assay kit (BC0305) were purchased from Solarbio. L-Glutamine (HY-N0390), Alpha-Ketoglutaric acid (HY-W013636), metformin (HY-B0627), CB-839 (HY-12248), and R162 (HY-103096) were purchased from MCE. Immunohistochemistry kit (abs996) and tryptophan microplate assay kit (abs580221) were purchased from Absin. Super-Enhanced Cell Counting Kit-8 (C0048S) was purchased from Beyotime.

Anti-β-actin monoclonal antibody (YM3028) was purchased from Immunoway. Anti-IFNAR1 monoclonal antibody (A0575) was purchased from ABclonal. FITC anti-mouse IgG antibody (406001) was purchased from Biolegend. Anti-GLS1 monoclonal antibody (66265-1-lg), Anti-GLUD1 polyclonal antibody (14299-1-AP), Anti-GOT1 polyclonal antibody (14886-1-AP), Anti-GOT2 monoclonal antibody (67738-1-lg), Anti-SLC1A5 polyclonal antibody (20350-1-AP), Anti-GS monoclonal antibody (66323-1-lg), FITC-conjugated goat anti-rabbit lgG (H+L) (SA00003-2) and CoraLite594-conjugated goat anti-mouse IgG (H+L) (SA00013-3) were purchased from Proteintech. Anti-GPT2 polyclonal antibody (ab232963) was purchased from Abcam. Anti-PEDV N protein monoclonal antibody was kindly provided by Prof. Guangxing Li from Northeast Agricultural University. Anti-RoVA VP6 protein polyclonal antibody was prepared by our laboratory.

### RNA extraction and RT-qPCR

Total RNA from intestine tissues and cells was extracted using the TRIzol reagent (Invitrogen, 15596026) and reverse transcription was performed with HiScript Q RT SuperMix for qPCR (Vazyme, R122-01). Quantitative real-time RT-PCR was performed using ABScript II One Step SYBR Green RT-qPCR Kit (ABclonal, RK20404) with QuantStudio 6 Flex Real-Time PCR System (Life Technologies). The expression levels of target genes were normalized to GAPDH. To quantify viral copies in the intestine tissues of piglets, 10-fold serial dilutions of plasmids containing viral protein (1 ×10^1^ to 1 × 10^8^ template copies per reaction) were analyzed by RT-qPCR, and the viral copies of PEDV or PoRVA were calculated based on the Ct values using the resulting standard curves. All primers used for RT-qPCR in this study are listed in S2 Table.

### Plaque assay

The plaque assay of PoRVA was performed as described previously [55]. Briefly, monolayers of MA104 cells were seeded in a 6-well plate, and infected with 5 μg/mL trypsin-pretreated PoRVA (RVA-SXXA or RVA-HNNY). After inoculation for 2 h, the cells were washed with PBS, and overlaid with 2% sodium carboxymethylcellulose and 2×M199 (containing 2% FBS and 5 μg/mL trypsin) at a 1:1 ratio. Plaques were fixed with 4% paraformaldehyde at day 3 to day 5 post-inoculation. The fixed samples were stained with 0.5% crystal violet at room temperature for 15 min for plaque visualization. Infectious titers of PEDV were determined with plaque assays as described in our previous study [54].

### Flow cytometry

For flow cytometry of virus-infected cells, we used the following procedure. IPEC-J2 cells were seeded in 6-well plates at a density of 1 × 10^6^ cells per well and grown for 24 hours. Cells were infected with PoRVA-SXXA or PoRVA-HNNY at 1 MOI for 12 h, then infected with PEDV (MOI = 1) for another 18 h followed by flow cytometry analysis. cells were harvested and washed twice with cold PBS, and then applied to immunostaining using an anti-PEDV N protein mAb to measure intracellular PEDV. The mock-infected cells were used for gating. The infection rate of PEDV were detected by FACs for fluorescent and data were analyzed by FlowJo software (Version 10).

### Western blotting and immunofluorescence

Western blot analysis was performed as described previously [56]. Briefly, cell lysates were harvested at the indicated time in RIPA buffer complemented with protease inhibitor mixture and then centrifuged at 12,000 g for 20 min at 4°C. The target proteins were separated on a 12% acrylamide gel and transferred onto PVDF membranes. The membranes were blocked in PBS buffer with 5% bovine serum albumin (BSA) for 2 h at room temperature, followed by incubated with the indicated primary antibodies diluted in PBS containing at 4°C overnight, and then incubation with the HRP-conjugated secondary antibody (Proteintech) at room temperature for 2 h. Protein bands were visualized with enhanced chemiluminescence (ECL) reagents (Bio-Rad).

The immunostaining was performed as described earlier. Briefly, IPEC-J2 cells grown on coverslips in 24-well culture plates were infected with RVA-SXXA or RVA-HNNY at 1 MOI for 12 h. Then the cells were washed with PBS, fixed with 4% paraformaldehyde for 20 min and permeabilized with 0.1% Triton X-100 for 15 min at room temperature. The cells were blocked with 2% BSA for 1 h. Primary antibodies were incubated for 1 h at room temperature. After washing three times, the cells were following incubated with secondary antibodies for 1 h at room temperature. After quick staining with DAPI, coverslips were mounted onto microscope slides in the presence of a fluorescence mounting medium. Samples were analyzed by Leica TCS SP8 laser scanning confocal microscope. Images were recorded using Leica X software.

### Animal experiment

Five-day-old suckling piglets were used in each animal experiment. All piglets were negative for PEDV, TGEV, PoRVA/B/C/H, SADS-CoV, PKV, PSV, PDCoV, PBoV, PEC (porcine enteric calicivirus), NoVs (porcine noroviruses), PSaV (porcine sapovirus), PAstV (porcine astrovirus), and other major swine pathogens as determined by PCR.

For the first animal experiment presented in Fig 1D–1F, twenty-four 5-day-old suckling piglets were randomly allocated to 4 groups of six piglets each and housed in different rooms. Piglets in the single-virus infection groups were orally inoculated with 4 × 10^5^ PFU of PEDV or 4 mL of DMEM (mock infection). Piglets in the coinfection groups were orally inoculated with 4 × 10^5^ PFU PoRVA (RVA-SXXA) or 4 × 10^5^ PFU PKV for 12 hours and then orally inoculated with PEDV (10^5^ PFU/mL). After inoculation, the clinical signs, including depression, slowing down, diarrhea, vomiting, and anorexia, of the piglets in each group were observed and recorded daily. Grading standards of clinical signs were as follows: 0 = normal; 1 = mild lethargy; 2 = moderate lethargy; 3 = heavier lethargy; and 4 = severe lethargy. The criteria for determining the fecal score were as follows: 0, normal; 1, soft (cowpie); 2, very soft and tends to be liquid; 3, liquid with some solid content; and 4, watery diarrhea with no solid content.

For the second animal experiment presented in Figs 2B and S3A–S3E, thirty-six 5-day-old suckling piglets were randomly allocated to six groups of six piglets each and housed in different rooms. Piglets in the single virus infection group were orally inoculated with 4 × 10^5^ PFU of RVA-SXXA, 4 × 10^5^ PFU of RVA-HNNY, 4 × 10^5^ PFU of PEDV or 4 mL of DMEM (mock infection). Piglets in the coinfection groups were orally inoculated with 4 × 10^5^ PFU RVA-SXXA or RVA-HNNY for 12 hours and then orally inoculated with PEDV (4 × 10^5^ PFU). After challenge, the clinical signs and diarrhea of the piglets in each group were observed and recorded daily. Rectal swabs were also collected on a daily basis for monitoring fecal viral RNA shedding by quantitative real-time RT–PCR targeting the PEDV N gene or PoRVA nsp5 gene. All of the surviving pigs were euthanized at the end of the animal experiments (7 days post-inoculation [dpi]).

For the third animal experiment presented in Figs 2C–2E and S3–S3H, eighteen 5-day-old suckling piglets were randomly allocated to six groups of three piglets each and housed in different rooms. The protocol used for the viral infection challenge experiments in piglets was the same as that used for the second animal experiment. To evaluate differences in the pathogenicity of each infection pattern in the acute phase of infection, all piglets were euthanized and dissected at 2 dpi to detect intestinal damage and the viral load in intestinal tissues in each group. The small intestinal tracts of the piglets were collected and fixed in 4% paraformaldehyde for 48 h. The fixed samples were subjected to hematoxylin and eosin (H&E) staining or immunohistochemistry (IHC) staining with a PoRVA VP6-specific polyclonal antibody or a PEDV N-specific monoclonal antibody. Histopathological examination and measurement of the jejunal villus height to crypt depth (VH:CD) ratios of each group of piglets were further performed. The intestinal tissue samples collected at necropsy were used to determine the distribution of PoRVA and PEDV in different segments of the intestinal tract by RT–qPCR.

### siRNA transfection

Specific siRNAs used to silence GLS1 and GLUD1 were listed in S2 Table. Specific siRNAs or negative control (NC) siRNA were transfected into IPEC-J2 cells using Lipofectamine 3000 transfection reagent (Thermo Fisher, L3000001) according to the manufacturer’s instructions. The effects of siRNAs were identified by Western blotting.

### Metabolite profiling

Virus-infected or mock-infected IPEC-J2 cells were used for global metabolic profiling. The IPEC-J2 cells from RVA-SXXA, RVA-HNNY, PEDV and mock groups were infected with RVA-SXXA (MOI 1), RVA-HNNY (MOI 1), PEDV (MOI 1), or 4 mL of DMEM (to simulate the infection), respectively. IPEC-J2 cells in the RVA-SXXA/PEDV group and RVA-HNNY/PEDV group were first infected with 1 MOI of RVA-SXXA or RVA-HNNY for 12 h, followed by PEDV for 12 h. Metabolites were extracted in ice-cold methanol, and endogenous metabolite profiles were obtained using LCMS methods as described previously [57]. The LC analysis was performed on a Vanquish UHPLC System (Thermo Fisher Scientific, USA). Chromatography was carried out with an ACQUITY UPLC HSS T3 (150 × 2.1 mm, 1.8 μm) (Waters, Milford, USA).

### Enzyme activity assay

The activities of GLS1, GLUD1, GOT, GPT2, GS were analyzed using the appropriate enzyme activity assay kits according to the manufacturer’s instructions. The cell samples were prepared as follows: the infected or mock-infected cells were first collected into centrifuge tubes, discard the supernatant, and 1mL of extraction solution was added per 5 × 10^6^ cells, followed by ultrasonic crushing of the cells. The cells were centrifuged at 3500 g for 10 min at 4°C, and the supernatant was removed and placed on ice for measurement. The enzyme activity was detected by using microplate reader at the specified wavelength.

### Cell viability and cell counting assay

The cell viability assays were performed using Super-Enhanced Cell Counting Kit-8 (Beyotime, C0048S) according to the manufacturer’s instructions. Results were expressed relative to those for control cells, defined as 100% viability. The cell growth numbers of WT cells and the impact of different doses of glutamine and arginine on cell growth numbers were assessed using Guava Muse Cell Analyzer (Luminex), according to the manufacturer’s instructions.

### Statistical analysis

Data are expressed as means ± SD and analyzed with SPSS 17.0. Differences between two experimental groups were performed using unpaired two-tailed Student’s t-tests. Comparisons among three or more experimental groups were performed using One-way analysis of variance (ANOVA). The survival of animals was compared using the log-rank (Mantel–Cox) test. Differences were considered to be statistically significant at * *P* < 0.05, ** *P* < 0.01. Statistical analysis was performed using GraphPad Prism 9 software (GraphPad Software, La Jolla, CA, USA).

## Supporting information

S1 Fig PoRVA and PEDV are the most common clinical porcine enteric viruses in Midwest China. **(A)** Geographical distribution of pig farms providing samples in four provinces of Midwest China. Map was plotted by the online platform (https://www.bioinformatics.com.cn). **(B)** The numbers of the 8 selected enteric viruses-positive pigs detected in the 582 diarrhea samples. The results are represented by a colored histogram; darker colors indicate multiple infections and shallower colors indicate single infections. **(C)** The numbers of other enteric virus-positive cases detected among PoRVA-positive pigs. **(D)** Statistical analysis of the correlations between PoRVA infection and infections caused by other pathogens. Odds ratios and 95% confidence intervals are presented to show the correlations of PoRVA with other viruses.
(TIF)

S2 Fig Virological features of RVA-HNNY and RVA-SXXA in vitro. **(A)** Phylogenetic trees based on the nucleotide sequence of VP7 or VP4 from RVA-SXXA, RVA-HMMY, and selected reference strains were constructed using the ML method in IQ-TREE v.2.1.2 software with 1000 bootstrap replicates. **(B)** Bright-field images of IPEC-J2 cells infected with RVA-SXXA or RVA-HNNY (MOI = 1) at 24 hpi. **(C)** PoRVA (RVA-SXXA and RVA-HNNY) infection in IPEC-J2 cells was detected by immunofluorescence. PoRVA VP6 (green), DAPI (blue). Scale bar, 100 μm. **(D)** The percentage of PoRVA-positive cells was determined based on three independent fractionation experiments performed as described in Panel (C). **(E)** IPEC-J2 cells were infected with RVA-SXXA or RVA-HNNY (MOI of 1) for 24 h. The infected cells were harvested for mRNA extraction. The copy number of the virus gene PoRVA (detected using nsp5 coding sequence-specific primers) was determined by RT–qPCR. **(F)** Comparison of the replication kinetics of two different subtypes of PoRVA in IPEC-J2 cells. Culture supernatants were harvested at the indicated times for detection of PoRVA titers by plaque assay.
(TIF)

S3 Fig PoRVA infection enhances the pathogenesis of PEDV in vivo. **(A-E)** The piglets in the single virus infection group were orally inoculated with RVA-SXXA (4 × 10^5^ PFU), RVA-HNNY (4 × 10^5^ PFU), PEDV (4 × 10^5^ PFU), or 4 mL of DMEM (mock infection) for 7 days. The piglets in the coinfection groups were orally inoculated with RVA-SXXA (4 × 10^5^ PFU) or RVA-HNNY (4 × 10^5^ PFU) for 12 hours and then orally inoculated with PEDV (4 × 10^5^ PFU) for 7 days. The survival of the piglets was monitored after PEDV inoculation for up to 7 days (A). The clinical significance score (B) and fecal score (C) for each group of piglets was observed and recorded daily. The criteria for the evaluation are described in the methods section. The average weight gain of the piglets was routinely measured. Each dot represents the average weight gain of an individual pig (D). The viral shedding of PoRVA was monitored up to 7 dpi by RT–qPCR targeting the nsp5 gene (E). **(F)** The viral loads of PoRVA in different intestinal segments of piglets as determined by quantitative RT–qPCR. **(G)** The viral titers in the jejunum of infected piglets from different groups were determined by plaque assays. **(H)** Histopathological and immunohistochemical images of the jejunum from challenged piglets collected 2 days after PEDV inoculation. Representative images of hematoxylin and eosin (H&E)-stained intestines (top row) and immunohistochemical staining of PEDV and PoRVA are shown in the middle and bottom rows, respectively. Red arrows and green arrows indicate the antigen distribution of PEDV and PoRVA, respectively, in intestinal tissues. **(I, J)** Replication and viral titers of RVA-HNNY and RVA-SXXA in IPEC-J2 cells were independent of PEDV infection. The viral replication of RVA-HNNY and RVA-SXXA was determined using RT–qPCR (I). The viral titers of PoRVA were detected by plaque assays (J).
(TIF)

S4 Fig PoRVA infection promotes PEDV replication in IPEC-J2 cells. IPEC-J2 cells were infected with RVA-SXXA or RVA-HNNY (MOI = 1) for 12 h, and then infected with PEDV (MOI = 1) for the indicated times. The infected cells were harvested for mRNA extraction, and PEDV gene copy numbers were quantified by RT–qPCR during the attachment, entry, and replication stages.
(TIF)

S5 Fig Stability and reliability analysis and metabolic pathway analysis of UPLC-MS/MS data. **(A)** Orthogonal projections to latent structures discriminant analysis (OPLS-DA) showed a clear separation between different groups and demonstrated the stability and reliability of the UPLC-MS/MS data. **(B)** The number of differentially abundant metabolites in the different groups compared to the mock group. Red, upregulated; blue, downregulated. **(C)** Bubble plots of the metabolic pathway enrichment analysis in PoRVA (left) or PEDV (right) -infected cells. Each bubble in the bubble diagram represents a metabolic pathway. The larger the bubble is, the more metabolites it contains. The x-axis represents a pathway impact value in the topology analysis, and the size is positively correlated with the influence factor. The y-axis represents the metabolic pathways identified in the enrichment analysis.
(TIF)

S6 Fig GLS1 and GLUD1 are involved in glutamine catabolism regulation during PoRVA infection. **(A, B)** The protein (A) and mRNA (B) expression of GLS1 and GLUD1 in si-GLS1- or si-GLUD1-transfected cells. The si-NC-transfected cells were used as controls. **(C-E)** IPEC-J2 cells were transfected with siRNA (si-GLS1 or si-GLUD1) and then mock-infected or infected with RVA-SXXA or RVA-HNNY (MOI of 1) for 12 h, followed by PEDV (MOI of 1) infection for another 12 h. The titers of PEDV were determined using a plaque formation assay (C). Cell lysates were harvested for the detection of PEDV N protein levels (D) and PEDV viral copies (E). **(F)** Metformin treatment suppressed the promotion of PEDV infection by PoRVA. IPEC-J2 cells were mock-infected or infected with RVA-SXXA or RVA-HNNY (MOI = 1) for 12 h, followed by infection with PEDV (MOI = 1) for another 12 h in the presence of metformin (5 mM). PEDV titers were determined using a plaque formation assay.
(TIF)

S1 Table Types of differentially expressed metabolites in this study. (DOCX)

S2 Table Primer sequence for RT-PCR, RT-qPCR, sgRNA, and siRNA. (DOCX)

S1 Data Data for Figs 1–5. (RAR)

S2 Data Data for Figs 6 and 7. (RAR)

S3 Data Data for Fig 8. (RAR)

S4 Data Data for S1 and S2 Figs. (RAR)

S5 Data Data for S3–S6 Figs. (RAR)
