# Supplementary material for: PoRVA G9P[23] and G5P[7] infections differentially promote PEDV replication by reprogramming glutamine metabolism
Source: PLoS Pathog. 2024 Jun 21;20(6):e1012305. doi: 10.1371/journal.ppat.1012305 (PMC11221755; doi:10.1371/journal.ppat.1012305)
Supplement: S1 Table — (DOCX) [file ppat.1012305.s007.docx]

**TABLE S1** Types of differentially expressed metabolites in this study.

| **RVA-SXXA vs. Mock（Total DE: 22, Up: 11, Down: 11）** | | | | | |
| --- | --- | --- | --- | --- | --- |
| **Metabolites** | **RVA-SXXA Mean** | **Mock Mean** | **FC** | **VIP** | **-log10 (P. value)** |
| cis-4-Hydroxy-L-proline | 56156252.56 | 15122802.74 | 3.71 | 2.16 | 1.87 |
| FAD | 1684458.91 | 597516.22 | 2.82 | 1.73 | 1.69 |
| (2R)-2-Hydroxy-3-(phosphonatooxy)propanoate | 9596017 | 4687369.07 | 2.05 | 1.92 | 2.36 |
| Undecanoic acid | 39469335.99 | 19961743.07 | 1.98 | 2.18 | 1.86 |
| Dodecanedioic acid | 1453366.03 | 1027091.79 | 1.42 | 1.57 | 1.32 |
| L-Arginine | 121310861.1 | 89936611.82 | 1.35 | 2.34 | 2.64 |
| D-Asparagine | 2801509.71 | 2093607.47 | 1.34 | 1.68 | 1.57 |
| 4'-Hydroxyacetophenone | 21867937.91 | 16702695.19 | 1.31 | 2.18 | 2.06 |
| L-beta-Phenylalanine | 1896641.09 | 1501286.1 | 1.26 | 2.08 | 1.33 |
| L-Glutamine | 41066956.25 | 32675821.58 | 1.26 | 2.15 | 1.92 |
| Pyroglutamic acid | 32248531.96 | 25670557.4 | 1.26 | 1.70 | 1.62 |
| 5-KETE | 2684947.53 | 2890799.85 | 0.93 | 1.82 | 2.02 |
| Phenyl acetate | 367736105.2 | 401601660.7 | 0.92 | 1.60 | 1.41 |
| Pipecolic acid | 172590903 | 191765531.9 | 0.90 | 1.99 | 1.55 |
| Eicosadienoic acid | 4194159.91 | 5359476.42 | 0.78 | 2.18 | 1.94 |
| myo-Inositol | 3725962.85 | 4749122.83 | 0.78 | 1.63 | 1.48 |
| L-Malic acid | 2771595.7 | 3643247.04 | 0.76 | 1.72 | 1.69 |
| Glyceric acid | 6642863.96 | 8772083.78 | 0.76 | 1.58 | 1.35 |
| Phthalic acid | 132758189.1 | 181134093.9 | 0.73 | 1.63 | 1.47 |
| N-Acetyl-a-neuraminic acid | 11982511.76 | 16398816.54 | 0.73 | 1.74 | 1.72 |
| Quinaldic acid | 10104642.74 | 13880498.21 | 0.73 | 2.03 | 1.6 |
| Pyrophosphate | 19469871.01 | 28176339.43 | 0.69 | 1.74 | 1.73 |
| **RVA-HNNY vs. Mock（Total DE: 43, Up: 32, Down: 11）** | | | | | |
| **Metabolites** | **RVA-HNNY Mean** | **Mock Mean** | **FC** | **VIP** | **-log10 (P. value)** |
| dTMP | 480507.9 | 80497.44 | 5.88 | 1.62 | 1.65 |
| Sedoheptulose 7-phosphate | 45670126.91 | 10573223 | 4.35 | 1.67 | 1.86 |
| Asymmetric dimethylarginine | 18359742.34 | 4917332 | 3.70 | 1.92 | 2.52 |
| 5'-Methylthioadenosine | 33966150.51 | 9125137 | 3.70 | 1.58 | 1.37 |
| Uridine diphosphate-N-acetylglucosamine | 35679603.55 | 10011555 | 3.57 | 1.67 | 2.57 |
| (2R)-2-Hydroxy-3-(phosphonatooxy)propanoate | 16132991.39 | 4687369 | 3.45 | 1.79 | 3.93 |
| 3-Phosphonopyruvate | 2763834.19 | 819800.4 | 3.33 | 2.06 | 3.73 |
| Terephthalate | 2609423.67 | 903050.3 | 2.86 | 1.80 | 4.17 |
| Octadecanamide | 434173250.5 | 1.68E+08 | 2.56 | 1.57 | 1.42 |
| L-Tryptophan | 3193827.49 | 1248670 | 2.56 | 1.63 | 2.39 |
| 5-Guanidino-3-methyl-2-oxopentanoate | 1870251.96 | 765953.4 | 2.44 | 1.65 | 1.59 |
| Dodecanoic acid | 2763792.22 | 1130835 | 2.44 | 1.49 | 1.79 |
| Capric acid | 829838.73 | 340784.7 | 2.44 | 2.11 | 5.14 |
| D-Asparagine | 4965473.09 | 2093607 | 2.38 | 1.81 | 4.23 |
| L-Glutamine | 206196922.3 | 89936612 | 2.27 | 2.10 | 4.4 |
| L-Arginine | 74382783.11 | 32675822 | 2.27 | 2.09 | 4.34 |
| D-Ribose | 84615172.76 | 39141707 | 2.17 | 1.98 | 2.97 |
| Coumarin | 32847735.47 | 15742011 | 2.08 | 1.35 | 1.37 |
| L-Tyrosine | 14278768.76 | 6879031 | 2.08 | 1.99 | 3.02 |
| Uridine | 20682228.04 | 10367771 | 2.00 | 1.60 | 2.22 |
| Phenylacetaldehyde | 44229813.25 | 22344094 | 1.96 | 2.13 | 6.22 |
| 4'-Hydroxyacetophenone | 28648640.71 | 16702695 | 1.72 | 2.00 | 3.13 |
| Dodecanedioic acid | 3462073.54 | 2031689 | 1.69 | 1.40 | 1.48 |
| Cholesterol sulfate | 1744542.5 | 1027092 | 1.69 | 1.61 | 2.28 |
| Guanosine-5'-triphosphate | 15910814.23 | 9744514 | 1.64 | 1.43 | 1.59 |
| 5-Aminosalicylate | 34118171.31 | 23280714 | 1.47 | 1.92 | 2.54 |
| 3-Hydroxyphenylacetic acid | 39499112.22 | 28415570 | 1.39 | 1.94 | 2.71 |
| Deoxyinosine | 9068035.81 | 7223730 | 1.25 | 1.58 | 2.08 |
| Adenosine | 2136168.17 | 1816169 | 1.18 | 1.67 | 2.57 |
| 2-Heptanone | 23156470.34 | 20538115 | 1.12 | 1.58 | 1.44 |
| D-Arabinose 5-phosphate | 254360.13 | 228505.9 | 1.11 | 1.38 | 1.44 |
| Citric acid | 9208697.04 | 8425466 | 1.10 | 1.42 | 1.56 |
| Sebacic acid | 18165585.54 | 19600189 | 0.93 | 1.72 | 1.74 |
| 3-Methyl-L-tyrosine | 4002659.99 | 4816703 | 0.83 | 1.55 | 1.34 |
| gamma-Aminobutyric acid | 4805906.93 | 6449183 | 0.75 | 1.60 | 2.19 |
| gamma-Glutamylcysteine | 242488536.8 | 3.52E+08 | 0.69 | 1.32 | 1.3 |
| L-4-Hydroxyphenylglycine | 40655735.54 | 60505879 | 0.67 | 1.81 | 2.04 |
| trans-1,2-Cyclohexanediol | 28579587.61 | 70935877 | 0.40 | 1.33 | 1.33 |
| Iminoarginine | 5498651.54 | 15436546 | 0.36 | 2.00 | 2.15 |
| Caproic acid | 179635610.4 | 6.52E+08 | 0.28 | 1.47 | 1.71 |
| Alpha-Tocotrienol | 893236.16 | 4720608 | 0.19 | 1.88 | 2.38 |
| 7-Dehydrocholesterol | 801444.65 | 6037834 | 0.13 | 1.58 | 1.43 |
| Deoxyuridine | 654938.09 | 7293642 | 0.09 | 2.13 | 5.96 |
| **RVA-HNNY vs. RVA-SXXA（Total DE: 43, Up: 33, Down: 10）** | | | | | |
| **Metabolites** | **RVA-HNNY Mean** | **RVA-SXXA Mean** | **FC** | **VIP** | **-log10 (P. value)** |
| Sedoheptulose 7-phosphate | 45670126.91 | 8310665.14 | 5.56 | 1.68 | 1.93 |
| dTMP | 480507.9 | 98534.23 | 4.76 | 1.53 | 1.43 |
| 5'-Methylthioadenosine | 33966150.51 | 9499003.91 | 3.57 | 1.54 | 1.31 |
| Uridine diphosphate-N-acetylglucosamine | 35679603.55 | 12234815.44 | 2.94 | 1.69 | 2.74 |
| Asymmetric dimethylarginine | 18359742.34 | 6935702.49 | 2.63 | 2.11 | 4.01 |
| Terephthalate | 2609423.67 | 1026793.43 | 2.56 | 1.72 | 2.99 |
| Pyridoxine | 3489164.58 | 1401163.6 | 2.50 | 1.92 | 1.79 |
| Capric acid | 2763792.22 | 1102293.96 | 2.50 | 1.51 | 1.87 |
| Cholesterol sulfate | 3462073.54 | 1551267.56 | 2.22 | 1.63 | 2.41 |
| Urocanic acid | 4633384.1 | 2251039.21 | 2.04 | 1.33 | 1.33 |
| Uridine | 20682228.04 | 10113221.73 | 2.04 | 1.76 | 3.5 |
| Phenylacetaldehyde | 44229813.25 | 22658043.33 | 1.96 | 2.16 | 5.25 |
| 3-Phosphonopyruvate | 2763834.19 | 1410158.9 | 1.96 | 1.72 | 1.65 |
| Dibutyl phthalate | 56510272.51 | 29077600.31 | 1.96 | 1.94 | 2.4 |
| Oleic acid | 728293053.8 | 391384399.5 | 1.85 | 1.73 | 1.33 |
| L-Glutamine | 74382783.11 | 41066956.25 | 1.82 | 2.13 | 4.25 |
| D-Asparagine | 4965473.09 | 2801509.71 | 1.79 | 1.78 | 3.66 |
| 5-Aminopentanoic acid | 16871563.6 | 9661530.66 | 1.75 | 1.80 | 4.13 |
| Pyrophosphate | 34147873.29 | 19469871.01 | 1.75 | 1.73 | 3.2 |
| L-Arginine | 206196922.3 | 121310861.1 | 1.69 | 2.11 | 3.9 |
| (2R)-2-Hydroxy-3-(phosphonatooxy)propanoate | 16132991.39 | 9596017 | 1.69 | 1.50 | 1.82 |
| Coumarin | 14278768.76 | 8749897.07 | 1.64 | 1.96 | 2.57 |
| L-Serine | 3418544.09 | 2191132.33 | 1.56 | 1.79 | 3.96 |
| Adrenic acid | 71753537.05 | 48284444.75 | 1.49 | 1.59 | 2.2 |
| 5-Aminosalicylate | 34118171.31 | 24446444.88 | 1.39 | 1.62 | 1.41 |
| Deoxyinosine | 9068035.81 | 6691899.21 | 1.35 | 1.40 | 1.5 |
| 4'-Hydroxyacetophenone | 28648640.71 | 21867937.91 | 1.32 | 1.90 | 2.31 |
| N-Acetyl-a-neuraminic acid | 15745754.36 | 11982511.76 | 1.32 | 1.40 | 1.53 |
| Saccharopine | 10010044.03 | 7711891.12 | 1.30 | 1.56 | 1.34 |
| Adenosine | 2136168.17 | 1710716.73 | 1.25 | 1.73 | 3.16 |
| Phthalic acid | 162808772 | 132758189.1 | 1.22 | 1.43 | 1.61 |
| Guanine | 127553119.8 | 109575338.4 | 1.16 | 1.60 | 2.27 |
| Pterin | 135666644.7 | 128378281.2 | 1.05 | 1.69 | 1.59 |
| gamma-Aminobutyric acid | 4805906.93 | 6267410.41 | 0.77 | 1.60 | 2.22 |
| Arachidonic acid | 14891364.94 | 23413405.72 | 0.64 | 1.40 | 1.53 |
| Allocholic acid | 6483390.51 | 12219717.56 | 0.53 | 1.66 | 1.53 |
| Phosphoenolpyruvic acid | 4957089.41 | 10747443.31 | 0.46 | 1.56 | 2.05 |
| L-Threonine | 13926136.22 | 33611495.86 | 0.41 | 1.66 | 1.56 |
| Dihydrouracil | 1446053.85 | 5672463.8 | 0.26 | 2.10 | 3.67 |
| cis-4-Hydroxy-L-proline | 13187410.46 | 56156252.56 | 0.23 | 1.85 | 2.06 |
| Caproic acid | 179635610.4 | 904811713.8 | 0.20 | 1.75 | 3.33 |
| Phenylpyruvic acid | 1710146.9  7 | 9069465.73 | 0.19 | 2.11 | 3.89 |
| 7-Dehydrocholesterol | 801444.65 | 5344875.16 | 0.15 | 1.73 | 1.72 |
| **PEDV vs. Mock（Total DE: 71, Up: 63, Down: 8）** | | | | | |
| **Metabolites** | **PEDV Mean** | **Mock Mean** | **FC** | **VIP** | **-log10 (P. value)** |
| Asymmetric dimethylarginine | 188857699.4 | 4917331.52 | 33.33 | 2.02 | 3.29 |
| dTMP | 3058619.87 | 80497.44 | 33.33 | 1.46 | 2.48 |
| Sedoheptulose 1,7-bisphosphate | 5229545.39 | 172214.95 | 33.33 | 1.47 | 2.55 |
| Galactose 1-phosphate | 48970697.93 | 1915289.86 | 25.00 | 1.40 | 2.83 |
| Sedoheptulose 7-phosphate | 163548557 | 10573222.9 | 16.67 | 1.48 | 2.58 |
| Uridine | 138902930.1 | 10367770.84 | 14.29 | 1.50 | 4.64 |
| Vanillylmandelic acid | 12499115.38 | 972785.07 | 12.50 | 1.78 | 1.65 |
| Inosine | 30115735.25 | 2409599.33 | 12.50 | 1.45 | 2.28 |
| 3-Phosphonopyruvate | 8919442.43 | 819800.4 | 11.11 | 1.84 | 1.89 |
| 5'-Methylthioadenosine | 101968324.5 | 9125137.3 | 11.11 | 1.87 | 2.69 |
| (2R)-2-Hydroxy-3-(phosphonatooxy)propanoate | 51192434.5 | 4687369.07 | 11.11 | 1.52 | 5.69 |
| 5-Guanidino-3-methyl-2-oxopentanoate | 6873977.55 | 765953.42 | 9.09 | 1.95 | 2.47 |
| Linoleic acid | 191548115.6 | 23511205.09 | 8.33 | 1.87 | 2.77 |
| Monomethyl sulfate | 64347858.22 | 8337269.25 | 7.69 | 1.78 | 2.23 |
| L-Tryptophan | 9949754.92 | 1248669.99 | 7.69 | 1.49 | 4.38 |
| Uridine diphosphate-N-acetylglucosamine | 78182514.32 | 10011554.81 | 7.69 | 1.47 | 3.67 |
| D-Asparagine | 13570990.09 | 2093607.47 | 6.67 | 1.52 | 6 |
| gamma-Amino-gamma-cyanobutanoate | 6411067.63 | 1019481.69 | 6.25 | 1.77 | 2.18 |
| Dibutyl phthalate | 292253973.5 | 49101769.79 | 5.88 | 1.90 | 3.03 |
| L-Serine | 8962959.4 | 1556576.1 | 5.88 | 1.39 | 1.88 |
| 5-Aminopentanoic acid | 38453305.21 | 6580297.85 | 5.88 | 1.39 | 1.87 |
| Phenylacetaldehyde | 123275381.1 | 22344093.55 | 5.56 | 2.06 | 8.66 |
| Creatine | 136648375.7 | 23962951.83 | 5.56 | 1.79 | 1.67 |
| Coumarin | 37520012.87 | 6879031.48 | 5.56 | 2.04 | 5.69 |
| Dodecanoic acid | 1938497.93 | 340784.65 | 5.56 | 2.04 | 5.41 |
| D-Arabinose 5-phosphate | 1267756 | 228505.87 | 5.56 | 1.35 | 1.74 |
| L-Arginine | 454521042.2 | 89936611.82 | 5.00 | 2.05 | 6.68 |
| Dodecanedioic acid | 5185767.66 | 1027091.79 | 5.00 | 1.51 | 4.89 |
| 4'-Hydroxyacetophenone | 80714972.89 | 16702695.19 | 4.76 | 2.04 | 5.72 |
| L-Glutamine | 151045915 | 32675821.58 | 4.55 | 2.05 | 6.52 |
| 3-Hydroxyphenylacetic acid | 111834981.7 | 28415569.55 | 4.00 | 2.04 | 5.73 |
| 4-Quinolinecarboxylic acid | 11479716.51 | 2902212.92 | 4.00 | 1.53 | 4.12 |
| FAD | 2377535.99 | 597516.22 | 4.00 | 1.13 | 1.43 |
| Pyridoxine | 8462452.6 | 2214675.35 | 3.85 | 1.45 | 1.31 |
| NAD | 2197230.29 | 611757.42 | 3.57 | 1.52 | 4.01 |
| AMP | 4592618.59 | 1350706.3 | 3.45 | 1.48 | 1.4 |
| L-Malic acid | 12279965.04 | 3643247.04 | 3.33 | 1.51 | 4.94 |
| Terephthalate | 2976868.04 | 903050.34 | 3.33 | 1.50 | 4.61 |
| UMP | 32825440.99 | 9733911.35 | 3.33 | 1.34 | 2.38 |
| N5-(L-1-Carboxyethyl)-L-ornithine | 2793549.74 | 877569.62 | 3.23 | 1.58 | 1.6 |
| Ricinoleic acid | 3170339.84 | 981740.22 | 3.23 | 1.14 | 1.47 |
| Cholesterol sulfate | 6349683.82 | 2031688.97 | 3.13 | 1.44 | 3.33 |
| 5-Aminosalicylate | 68465414.76 | 23280713.64 | 2.94 | 1.71 | 1.53 |
| Capric acid | 3295542.73 | 1130835.13 | 2.94 | 1.31 | 2.16 |
| Epiandrosterone | 112203802.3 | 38166305.4 | 2.94 | 1.40 | 2.84 |
| 2-Heptanone | 59521708.37 | 20538115.36 | 2.86 | 2.02 | 4.73 |
| Propanoyl phosphate | 288228505 | 99656381.96 | 2.86 | 1.86 | 1.92 |
| (R)-4-Hydroxymandelate | 5404159.59 | 1900716.78 | 2.86 | 1.69 | 1.91 |
| Adrenic acid | 153156128.5 | 60479495.42 | 2.56 | 1.47 | 3.84 |
| Phenylacetic acid | 20160207.86 | 8292705.45 | 2.44 | 1.95 | 3.31 |
| Desmosterol | 20772287.81 | 8808892.31 | 2.38 | 1.63 | 1.68 |
| L-beta-Phenylalanine | 3438965.74 | 1501286.1 | 2.27 | 1.93 | 3.27 |
| (R) 2,3-Dihydroxy-3-methylvalerate | 2021681.21 | 986496.23 | 2.04 | 1.52 | 1.48 |
| allopurinol  Selenomethionine | 36011215.6 | 17791727.04 | 2.04 | 1.57 | 1.61 |
| o-Toluate | 3584700.01 | 1773175.24 | 2.04 | 1.11 | 1.38 |
| allopurinol | 111711858.7 | 57008907.87 | 1.96 | 1.76 | 2.16 |
| Phenyl acetate | 718065429.4 | 401601660.7 | 1.79 | 1.51 | 5.07 |
| Pyrophosphate | 46810449.04 | 28176339.43 | 1.67 | 1.38 | 2.64 |
| Undecanoic acid | 32538264.46 | 19961743.07 | 1.64 | 1.58 | 1.66 |
| Tryptophanol | 1902237.82 | 1362645.29 | 1.39 | 1.48 | 1.31 |
| Phthalic acid | 251342884 | 181134093.9 | 1.39 | 1.24 | 1.41 |
| Folic acid | 1520245.07 | 1128967.17 | 1.35 | 1.39 | 2.73 |
| Corticosterone | 11257666.99 | 10266607.04 | 1.10 | 1.16 | 1.53 |
| Uracil | 15024798.16 | 17512296.93 | 0.85 | 1.11 | 1.38 |
| Iminoarginine | 11642617.02 | 15436546.22 | 0.75 | 1.92 | 3.09 |
| Pentadecanoic acid | 7432666.86 | 10546795.12 | 0.70 | 1.27 | 1.94 |
| alpha-Spinasterol | 20727824.38 | 35129185.39 | 0.59 | 1.46 | 1.33 |
| Mirtazapine | 5249870 | 9655523.9 | 0.54 | 1.43 | 1.33 |
| O-Phosphoethanolamine | 166176363 | 458094390.4 | 0.36 | 1.75 | 2.08 |
| 12-Keto-leukotriene B4 | 1001699.88 | 4625469.17 | 0.22 | 1.33 | 2.27 |
| Erucic acid | 510240114.4 | 4950902303 | 0.10 | 1.73 | 2.06 |
| **RVA-SXXA/PEDV vs. PEDV（Total DE: 24, Up: 12, Down: 12）** | | | | | |
| **Metabolites** | **RVA-SXXA/PEDV Mean** | **PEDV Mean** | **FC** | **VIP** | **-log10 (P. value)** |
| (S)-2,3-Epoxysqualene | 62845396 | 18692054 | 3.36 | 1.81 | 1.67 |
| 12-Keto-leukotriene B4 | 2975891 | 1001700 | 2.97 | 1.86 | 2.17 |
| 3-Phosphoglyceric acid | 44095864 | 14954976 | 2.95 | 1.72 | 1.48 |
| AMP | 10261675 | 4592619 | 2.23 | 1.68 | 1.38 |
| Arachidic acid | 25642540 | 18846344 | 1.36 | 1.73 | 1.83 |
| UDP | 3570377 | 2670263 | 1.34 | 1.53 | 1.33 |
| 3-Hydroxyphenylacetic acid | 1.47E+08 | 1.12E+08 | 1.32 | 1.99 | 2.24 |
| N-Formyl-L-methionine | 26964041 | 21328772 | 1.26 | 1.79 | 1.59 |
| Sedoheptulose 7-phosphate | 1.97E+08 | 1.64E+08 | 1.2 | 1.80 | 2 |
| Deoxyinosine | 8734166 | 7581959 | 1.15 | 1.84 | 2.2 |
| Phenylacetaldehyde | 1.38E+08 | 1.23E+08 | 1.12 | 1.90 | 1.95 |
| Adenosine | 2003153 | 1795266 | 1.12 | 1.53 | 1.31 |
| Nicotine | 44234448 | 51241744 | 0.86 | 1.87 | 1.78 |
| Dodecanedioic acid | 4052719 | 5185768 | 0.78 | 1.73 | 1.8 |
| Folic acid | 1089060 | 1520245 | 0.72 | 1.87 | 2.23 |
| D-Phenyllactic acid | 10541894 | 14846556 | 0.71 | 1.64 | 1.37 |
| 4a-Carbinolamine tetrahydrobiopterin | 26585457 | 38978592 | 0.68 | 1.99 | 2.24 |
| Guanosine | 1.1E+08 | 1.64E+08 | 0.67 | 2.06 | 2.43 |
| Ethylmethylacetic acid | 13955609 | 24256044 | 0.58 | 1.59 | 1.49 |
| Methyl beta-D-galactoside | 5875319 | 11468186 | 0.51 | 1.80 | 1.6 |
| Norepinephrine | 37143527 | 86106130 | 0.43 | 1.74 | 1.48 |
| Pelargonic acid | 1053851 | 2618077 | 0.4 | 1.54 | 1.32 |
| Desmosterol | 7880364 | 20772288 | 0.38 | 1.91 | 1.88 |
| N,N-Diethyl-m-toluamide | 6784476 | 26564428 | 0.26 | 1.72 | 1.46 |
| **RVA-HNNY/PEDV vs. PEDV（Total DE: 42, Up: 24, Down: 18）** | | | | | |
| **Metabolites** | **RVA-HNNY/PEDV Mean** | **PEDV Mean** | **FC** | **VIP** | **-log10 (P. value)** |
| Erucic acid | 4.21E+09 | 5.1E+08 | 8.25 | 1.89 | 1.81 |
| gamma-Glutamyl-beta-aminopropiononitrile | 2321248 | 860239.8 | 2.7 | 1.83 | 1.52 |
| gamma-Amino-gamma-cyanobutanoate | 15949077 | 6411068 | 2.49 | 1.79 | 1.43 |
| Monomethyl sulfate | 1.6E+08 | 64347858 | 2.48 | 1.76 | 1.38 |
| Dibutyl phthalate | 6.18E+08 | 2.92E+08 | 2.11 | 2.00 | 1.9 |
| Uridine | 2.91E+08 | 1.39E+08 | 2.09 | 1.58 | 1.48 |
| 24-Methylenecycloartanol | 5612838 | 3061520 | 1.83 | 1.86 | 1.72 |
| CMP | 6895049 | 3965224 | 1.74 | 1.54 | 1.41 |
| GMP | 7803303 | 4660414 | 1.67 | 1.87 | 1.74 |
| dTMP | 4863444 | 3058620 | 1.59 | 1.76 | 1.98 |
| Dodecanoic acid | 2957207 | 1938498 | 1.53 | 2.10 | 1.8 |
| Iminoarginine | 15951810 | 11642617 | 1.37 | 2.00 | 1.96 |
| L-Tryptophan | 13542258 | 9949755 | 1.36 | 1.70 | 1.79 |
| L-Arginine | 6.12E+08 | 4.55E+08 | 1.35 | 2.29 | 3.73 |
| L-Glutamine | 2.02E+08 | 1.51E+08 | 1.34 | 2.33 | 4.46 |
| Coumarin | 47782905 | 37520013 | 1.27 | 2.22 | 2.92 |
| Phenylacetaldehyde | 1.55E+08 | 1.23E+08 | 1.26 | 2.11 | 2.32 |
| 5-Aminopentanoic acid | 46797814 | 38453305 | 1.22 | 1.79 | 2.03 |
| Asymmetric dimethylarginine | 2.27E+08 | 1.89E+08 | 1.2 | 1.76 | 1.44 |
| Deoxyinosine | 8852074 | 7581959 | 1.17 | 1.70 | 1.79 |
| Sedoheptulose 7-phosphate | 1.89E+08 | 1.64E+08 | 1.16 | 1.58 | 1.51 |
| 4-Quinolinecarboxylic acid | 13207855 | 11479717 | 1.15 | 1.57 | 1.45 |
| Phosphonoacetate | 3379713 | 3045776 | 1.11 | 1.57 | 1.45 |
| 2',4'-Dihydroxyacetophenone | 1752353 | 1619801 | 1.08 | 1.51 | 1.34 |
| Cytidine | 6555586 | 7123088 | 0.92 | 1.57 | 1.41 |
| Nicotinic acid | 49916303 | 55939772 | 0.89 | 1.70 | 1.32 |
| Pyrophosphate | 39013103 | 46810449 | 0.83 | 1.65 | 1.63 |
| Phthalic acid | 2.07E+08 | 2.51E+08 | 0.82 | 1.82 | 2.2 |
| 2-Heptanone | 47361828 | 59521708 | 0.8 | 1.77 | 1.45 |
| Folic acid | 1149838 | 1520245 | 0.76 | 1.89 | 2.51 |
| UMP | 24765033 | 32825441 | 0.75 | 1.63 | 1.58 |
| 4a-Carbinolamine tetrahydrobiopterin | 28338427 | 38978592 | 0.73 | 1.93 | 1.79 |
| Guanosine | 1.19E+08 | 1.64E+08 | 0.72 | 2.12 | 2.65 |
| 2-Aminobenzenesulfonate | 14242237 | 21292138 | 0.67 | 2.04 | 2.24 |
| allopurinol | 70957567 | 1.12E+08 | 0.64 | 1.73 | 1.35 |
| threo-3-Hydroxy-L-aspartate | 9497029 | 14848391 | 0.64 | 1.73 | 1.3 |
| Phenyl acetate | 4.15E+08 | 7.18E+08 | 0.58 | 1.99 | 3.24 |
| Ethylmethylacetic acid | 13810490 | 24256044 | 0.57 | 1.58 | 1.49 |
| Inosine | 16174303 | 30115735 | 0.54 | 1.77 | 1.47 |
| Uridine diphosphate-N-acetylglucosamine | 41222989 | 78182514 | 0.53 | 1.68 | 1.73 |
| (R) 2,3-Dihydroxy-3-methylvalerate | 1006532 | 2021681 | 0.5 | 1.87 | 1.58 |
| Creatinine | 23081182 | 54417257 | 0.42 | 1.91 | 1.74 |
| **RVA-HNNY/PEDV vs. RVA-SXXA/PEDV（Total DE: 48, Up: 26, Down: 22）** | | | | | |
| **Metabolites** | **RVA-HNNY/PEDV Mean** | **RVA-SXXA/PEDV Mean** | **FC** | **VIP** | **-log10 (P. value)** |
| Sphinganine | 65706923 | 17304540 | 3.8 | 2.23 | 4.57 |
| Galactose 1-phosphate | 1.44E+08 | 46137744 | 3.12 | 1.54 | 1.7 |
| Uridine | 2.91E+08 | 1.03E+08 | 2.83 | 1.71 | 2.32 |
| Dibutyl phthalate | 6.18E+08 | 2.19E+08 | 2.82 | 2.10 | 2.97 |
| Desmosterol | 21666202 | 7880364 | 2.75 | 2.15 | 3.43 |
| Monomethyl sulfate | 1.6E+08 | 59355260 | 2.69 | 1.83 | 1.79 |
| gamma-Amino-gamma-cyanobutanoate | 15949077 | 6152117 | 2.59 | 1.82 | 1.76 |
| 2-Oxo-4-methylthiobutanoic acid | 12650281 | 6307180 | 2.01 | 1.68 | 1.43 |
| Vanillylmandelic acid | 18593702 | 9701690 | 1.92 | 1.72 | 1.53 |
| Phosphoenolpyruvic acid | 8027912 | 4469721 | 1.8 | 1.43 | 1.4 |
| Iminoarginine | 15951810 | 10614984 | 1.5 | 1.93 | 2.08 |
| N-Acetylserotonin | 1.22E+08 | 83116109 | 1.46 | 1.75 | 1.57 |
| dTMP | 4863444 | 3345535 | 1.45 | 1.61 | 1.91 |
| D-Phenyllactic acid | 15167458 | 10541894 | 1.44 | 1.92 | 2.04 |
| (R)-4-Hydroxymandelate | 9933412 | 6941758 | 1.43 | 1.73 | 1.53 |
| Agmatine | 4932214 | 3563013 | 1.38 | 2.02 | 2.5 |
| L-Tryptophan | 13542258 | 9848417 | 1.38 | 1.71 | 2.34 |
| Coumarin | 47782905 | 35090713 | 1.36 | 2.08 | 2.85 |
| L-Arginine | 6.12E+08 | 4.58E+08 | 1.34 | 2.13 | 3.15 |
| N5-(L-1-Carboxyethyl)-L-ornithine | 4074931 | 3030044 | 1.34 | 1.75 | 1.57 |
| L-Glutamine | 2.02E+08 | 1.54E+08 | 1.31 | 2.14 | 3.29 |
| Tryptophanol | 1983524 | 1655556 | 1.2 | 1.72 | 1.52 |
| 5-Aminopentanoic acid | 46797814 | 39833322 | 1.17 | 1.57 | 1.77 |
| Creatine | 1.5E+08 | 1.33E+08 | 1.13 | 1.65 | 1.39 |
| L-Histidine | 19247158 | 16964530 | 1.13 | 1.61 | 1.91 |
| Nootkatone | 10036176 | 9291287 | 1.08 | 1.66 | 1.37 |
| Nonadecanoic acid | 1.9E+08 | 2.18E+08 | 0.87 | 1.51 | 1.58 |
| NAD | 1981349 | 2535034 | 0.78 | 1.43 | 1.38 |
| L-Asparagine | 48083830 | 62809884 | 0.77 | 1.69 | 1.46 |
| UDP | 2753977 | 3570377 | 0.77 | 1.43 | 1.38 |
| N-Acetyl-a-neuraminic acid | 15967106 | 20998381 | 0.76 | 1.54 | 1.66 |
| 2-Heptanone | 47361828 | 63480004 | 0.75 | 2.04 | 2.53 |
| Phytosphingosine | 6968851 | 9252071 | 0.75 | 2.01 | 2.43 |
| 3-Hydroxyphenylacetic acid | 1.08E+08 | 1.47E+08 | 0.73 | 2.08 | 2.79 |
| alpha-Tocopherol | 8930016 | 12360857 | 0.72 | 1.74 | 1.56 |
| Eicosadienoic acid | 4727144 | 6676909 | 0.71 | 1.88 | 1.91 |
| Quinaldic acid | 11769114 | 16843429 | 0.7 | 1.78 | 1.65 |
| N-Formyl-L-methionine | 18828965 | 26964041 | 0.7 | 1.77 | 1.62 |
| Pyrophosphate | 39013103 | 55831734 | 0.7 | 1.70 | 2.29 |
| 5-Aminosalicylate | 64856542 | 96858347 | 0.67 | 2.04 | 2.51 |
| (2Z,4S,5R)-2-Amino-4,5,6-trihydroxyhex-2-enoate | 8117124 | 14328703 | 0.57 | 1.77 | 1.62 |
| allopurinol | 70957567 | 1.29E+08 | 0.55 | 2.11 | 2.96 |
| Pseudouridine 5'-phosphate | 468998 | 858759.3 | 0.55 | 1.42 | 1.37 |
| Phenyl acetate | 4.15E+08 | 8E+08 | 0.52 | 1.79 | 2.82 |
| Naringin | 59562.21 | 145507.7 | 0.41 | 1.65 | 2.04 |
| 3-Phosphoglyceric acid | 15825015 | 44095864 | 0.36 | 1.87 | 1.91 |
| 4-Hydroxyphenylacetaldehyde | 6156512 | 18375344 | 0.34 | 1.80 | 1.69 |
| Guanidinosuccinic acid | 12552640 | 45592954 | 0.28 | 1.80 | 2.01 |
