# Supplementary material for: PoRVA G9P[23] and G5P[7] infections differentially promote PEDV replication by reprogramming glutamine metabolism
Source: PLoS Pathog. 2024 Jun 21;20(6):e1012305. doi: 10.1371/journal.ppat.1012305 (PMC11221755; doi:10.1371/journal.ppat.1012305)
Supplement: S2 Table — (DOCX) [file ppat.1012305.s008.docx]

**TABLE S2** Primer sequence for RT-PCR, RT-qPCR, sgRNA, and siRNA.

| Gene | Primers | Sequence (5’→3’) | Purpose |
| --- | --- | --- | --- |
| SLC1A5 | SLC1A5-F | AAGATGATCATCTTGCCGC | RT-qPCR |
|  | SLC1A5-R | AAGATCCAGGAACGAATCGAG |  |
| SLC38A1 | SLC38A1-F | CCTTCTGTTGATCTGTTCAA | RT-qPCR |
|  | SLC38A1-R | TTAGCTCTGGATCACTGCAG |  |
| SLC38A2 | SLC38A2-F | GCAGTGGAATCCTTGGGCTT | RT-qPCR |
|  | SLC38A2-R | TAAAGACCCTCCTTCATTGGC |  |
| SLC38A5 | SLC38A5-F | GTATGCTGTTTTTCCTCATTTCGG | RT-qPCR |
|  | SLC38A5-R | CTTGAGGGGCCCTTACTCTCC |  |
| SLC38A7 | SLC38A7-F | CTTCGTCTTCCCAGGTCTG | RT-qPCR |
|  | SLC38A7-R | GGTGACCAAGAGGATTCCG |  |
| SLC7A5 | SLC7A5-F | CCTGCCCGTGTTCTTCAT | RT-qPCR |
|  | SLC7A5-R | GCTGAGGATGATGGTGAA |  |
| SLC7A8 | SLC7A8-F | CAGGCAACGAAACAACACTG | RT-qPCR |
|  | SLC7A8-R | GAGCCGATGATGTTCCCTAC |  |
| SLC7A7 | SLC7A7-F | CCTTTGTTATGCGGAACTGG | RT-qPCR |
|  | SLC7A7-R | AAAGGTGATGGCAATGACCG |  |
| SLC7A6 | SLC7A6-F | CAGGACGCCTTTGAGGGTTC | RT-qPCR |
|  | SLC7A6-R | GAAATCCCAATGGCCAGAGG |  |
| SLC6A14 | SLC6A14-F | CTTGGTCTCGTCTGTGTGAC | RT-qPCR |
|  | SLC6A14-R | CTGTTCCCTCCATAAATCCAG |  |
| RVA-NSP5 | RVA-NSP5-F | AGCTCCACAATCTGAAGCAC | RT-qPCR |
|  | RVA-NSP5-R | CACGAATAATCAAATCCAGC |  |
| PEDV-N | PEDV-N-F | CTTTGGTGGTAATGTGGCTG | RT-qPCR |
|  | PEDV-N-R | TCAACAGCTGTGTCCCATTC |  |
| GAPDH | GAPDH-F | ACATCATCCCTGCTTCTACTGG | RT-qPCR |
|  | GAPDH-R | CTCGGACGCCTGCTTCAC |  |
| TGEV | TGEV-F | CTATCGCATGGTGAAGGGC | RT-PCR |
|  | TGEV-R | GGATCATCCTTTGGCAAGTG |  |
| PDCoV | PDCOV-F | GCCTTTACTGCTTGAATATGG | RT-PCR |
|  | PDCOV-R | CGCATCCTTAAGTCTCTC |  |
| SADS-CoV | SADS-F | ATGGCCACTGTTAATTGGG | RT-PCR |
|  | SADS-R | GAGCACGAGGTGTCTGAGC |  |
| PSV | PSV-F | CTCCACCCTTAAGGTGGTTG | RT-PCR |
|  | PSV-R | GTCGCCGAATCACCAAAGG |  |
| PKV | PKV-F | CTTCGGGACTGGTTTGGAGG | RT-PCR |
|  | PKV-R | GCAACCACAGTGCACTTC |  |
| PEDV | PEDV-F | TAGGACTCGTACTGAGGGTGT | RT-PCR |
|  | PEDV-R | CTATTTTCGCCCTTGGGAATT |  |
| PoRVA | PoRVA-F | GAAACGGAATAGCTCCACA | RT-PCR |
|  | PoRVA-R | GAATAATCAAATCCAGCCAC |  |
| PBoV | PBoV-F | CGAAGCGAGCGTCTAGGTAAG | PCR |
|  | PBoV-R | GGTTGAACGGACCCAAGTAT |  |
| SLC1A5 | SLC1A5-sg1-F | CACCGCGCCGCGAACGCCTTGGGGT | Knockout |
|  | SLC1A5-sg1-R | AAACACCCCAAGGCGTTCGCGGCGC |  |
|  | SLC1A5-sg2-F | CACCGGGCCGACCCCAAGGCGTTCG |  |
|  | SLC1A5-sg2-R | AAACCGAACGCCTTGGGGTCGGCCC |  |
|  | SLC1A5-sg3-F | AAACGGCCGAGACCCCGTTGGCGG |  |
|  | SLC1A5-sg3-R | AAACCCGCCAACGGGGTCTCGGCCC |  |
| IFNAR1 | IFNAR1-sg1-F | CACCGTCCGTGGGTGTTGCCCGCAG | Knockout |
|  | IFNAR1-sg1-R | AAACCTGCGGGCAACACCCACGGAC |  |
|  | IFNAR1-sg2-F | CACCGAGATTATCAAATAACAGGGA |  |
|  | IFNAR1-sg2-R | AAACTCCCTGTTATTTGATAATCTC |  |
|  | IFNAR1-sg3-F | CACCGCCATTTCAAGAAGCACAAAT |  |
|  | IFNAR1-sg3-R | AAACATTTGTGCTTCTTGAAATGGC |  |
| GLS1 | siGLS-1-F1 | GAUUUGCUGUUCUAUACAAUU | Knockdown |
|  | SiGLS1-R1 | UUGUAUAGAACAGCAAAUCUU |  |
|  | siGLS-1-F2 | GAGUGUAUGGAUAUGUUAAGA |  |
|  | siGLS-1-R2 | UUAACAUAUCCAUACACUCUU |  |
|  | siGLS-1-F3 | GAGUUAUAUGAAAGUGCUAAA |  |
|  | siGLS-1-R3 | UAGCACUUUCAUAUAACUCAU |  |
| GLUD1 | siGLUD1-F1 | GAACUAUACCGAUAAUGAAUU | Knockdown |
|  | siGLUD1-R1 | UUCAUUAUCGGUAUAGUUCUU |  |
|  | siGLUD1-F2 | AGCUAGUGGAGGACCUCAAGA |  |
|  | siGLUD1-R2 | UUGAGGUCCUCCACUAGCUUG |  |
|  | siGLUD1-F3 | AAGCUGGUGUUAAGAUCAACC |  |
|  | siGLUD1-R3 | UUGAUCUUAACACCAGCUUUA |  |
